# Inhibition of CDH11 Activates cGAS‐STING by Stimulating Branched Chain Amino Acid Catabolism and Mitigates Lung Metastasis of Adenoid Cystic Carcinoma

**DOI:** 10.1002/advs.202408751

**Published:** 2024-12-31

**Authors:** Rui‐Feng Li, Shuo Liu, Qian Gao, Min Fu, Xin‐Yi Sun, Mian Xiao, Xi‐Yuan Ge, Xin Peng

**Affiliations:** ^1^ Department of Oral and Maxillofacial Surgery Peking University School and Hospital of Stomatology Beijing 100081 P. R. China; ^2^ Central Laboratory Peking University School and Hospital of Stomatology Beijing 100081 P. R. China; ^3^ National Center for Stomatology Beijing 100081 P. R. China; ^4^ National Clinical Research Center for Oral Diseases Beijing 100081 P. R. China; ^5^ National Engineering Research Center of Oral Biomaterials and Digital Medical Devices BeiJing 100081 P. R. China

**Keywords:** adenoid cystic carcinoma, celecoxib, hybrid epithelial‐mesenchymal transforming cells, spatial transcriptome sequencing, single‐cell transcriptome sequencing, targeted therapy

## Abstract

Salivary adenoid cystic carcinoma (SACC) is an intractable malignant tumor originates in the secretory glands and frequently metastasizes to the lungs. Hybrid epithelial‐mesenchymal transition (EMT) cells within the tumors are correlated with augmented proliferative capacity and facilitation of lung metastasis. Single‐cell RNA sequencing and spatial transcriptomic sequencing are employed to reveal the hybrid EMT subsets within the vascular fibroblast microenvironment. These hybrid EMT cells exhibit a pro‐tumorigenic impact in vitro. Notably, cadherin 11 (CDH11), a specific marker for hybrid EMT cells, may exert its regulatory role in cellular function by interfering with branched‐chain amino acids (BCAA) metabolism by inhibiting branched‐chain ketoacid dehydrogenase to activate the mammalian target of the rapamycin pathway, thus making it a potential therapeutic target for SACC. Furthermore, celecoxib and its derivatives are specific CDH11 inhibitors that regulate BCAA metabolism, increase reactive oxygen species production, and subsequently activate the cyclic GMP‐AMP synthase‐stimulator of the interferongene pathway (cGAS‐STING). They also inhibit lung metastasis in NOD‐SCID mice in vivo. Overall, these findings suggest a promising treatment strategy that targets hybrid EMT cells to mitigate lung metastasis in SACC. Celecoxib may serve as a promising clinical intervention for the treatment of lung metastases in patients with SACC.

## Graphical abstract

The highly expressed CDH11 in hybrid EMT cells may be a potential therapeutic target for SACC. CXB and its derivatives, as specific inhibitors of CDH11, modulate BCAA metabolism, enhance ROS production, and subsequently activate the cGAS‐STING pathway. In vitro, celecoxib and its derivatives inhibit SACC lung metastasis in NOD‐SCID mice.

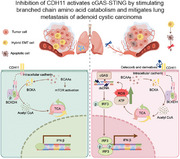

## 1 Introduction

Adenoid cystic carcinoma (ACC) is an intractable malignant epithelial cancer commonly found in the salivary glands of the head and neck, and salivary adenoid cystic carcinoma (SACC) accounts for 89.8% of all cases.^[^ ^1^ ^]^ It predominantly develops in the minor salivary glands,^[^ ^2^ ^]^ followed by the major salivary glands, including the parotid, submandibular gland (SMG), and sublingual salivary glands.^[^ ^3^ ^]^ ACC accounts for 25.2% of all malignant salivary gland tumors, making it the second most prevalent malignancy affecting the salivary glands.^[^ ^2^ ^]^ The natural course of ACC involves a slow growth, yet it is characterized by high recurrence rates, perineural invasion, and distant metastasis, particularly lung metastasis.^[^ ^1^, ^4^ ^]^ Consequently, the overall prognosis of ACC remains poor, with long‐term overall survival (OS) rates ranging from 23% to 40%.^[^ ^5^ ^]^ ACC is heterogeneous and can be classified into three pathological types: tubular, cribriform, and solid. The solid subtype is associated with higher relapse rates and earlier metastasis, leading to a generally unfavorable prognosis. Recent studies have further subdivided ACC into different subtypes (ACC‐I and ACC‐II) using genome sequencing techniques, RNA sequencing (RNA‐seq), and proteomic analyses.^[^ ^1^, ^6^ ^]^ However, owing to the lack of a comprehensive understanding of the molecular mechanisms underlying ACC, there is currently a shortfall of effective chemotherapy or targeted drugs for treating refractory tumors and preventing their metastasis to the lungs. Therefore, a deeper understanding of the molecular mechanisms governing the occurrence and development of ACC is crucial.

Lung metastasis is a critical determinant of patient prognosis. Epithelial‐mesenchymal transition (EMT) has been widely recognized as a key event in the initiation of tumor metastasis.^[^ ^7^, ^8^ ^]^ Studies have shown that the EMT process typically remains incomplete, resulting in cells exhibiting an intermediate state characterized by both epithelial and mesenchymal traits, commonly referred to as partial/hybrid EMT.^[^ ^9^ ^]^ Pastushenko et al. discovered that lung metastasis and proliferation were significantly enhanced in hybrid EMT cell subtypes that predominantly exhibited an epithelial cell phenotype.^[^ ^10^, ^11^ ^]^ Therefore, the identification and investigation of hybrid EMT cells in SACC can provide valuable insights into the mechanisms of lung metastasis and targeted treatment for lung metastasis.

With the advancements in transcriptome sequencing technology, single‐cell RNA sequencing (scRNA‐seq) has emerged as a powerful tool to unravel the cellular composition of tumors at single‐cell resolution and investigate both types of tumoral heterogeneity.^[^ ^12^, ^13^, ^14^, ^15^ ^]^ However, the lack of spatial information in scRNA‐seq hinders its ability to determine the spatial distribution in different cells. Spatial transcriptome sequencing (ST), on the other hand, can address these technical shortcomings and provide valuable insights into cellular location patterns. The integration of scRNA‐seq and ST enables mutual complementation, facilitating the acquisition of cellular heterogeneity and spatial information regarding the structural localization of cells within tissues. This is a pivotal strategy for investigating hybrid EMT cells in SACC.

In this study, we employed a combination of scRNA‐seq and ST to elucidate the cellular composition, functional characteristics, and spatial distribution of SACC. Owing to its high biological variability and lack of biomarkers for targeted therapy, SACC treatment remains a significant challenge.^[^ ^16^, ^17^ ^]^ Currently, targeted therapy is not available in clinical practice for patients with lung metastases. Our aim was to gain deeper insights into the molecular mechanisms of hybrid EMT state cells by combining scRNA‐seq and ST analysis and exploit their potential as therapeutic targets for mitigating lung metastasis in SACC. Therefore, we focused on elucidating the role of hybrid EMT cells in SACC and uncovering their therapeutic targets to enhance OS rates and improve patient quality of life.

## 2 Results

### 2.1 Infiltration of Immune Cells in Salivary adenoid cystic carcinoma is Comparatively Diminished, Particularly in Solid‐Type SACC

**Figure 1:**
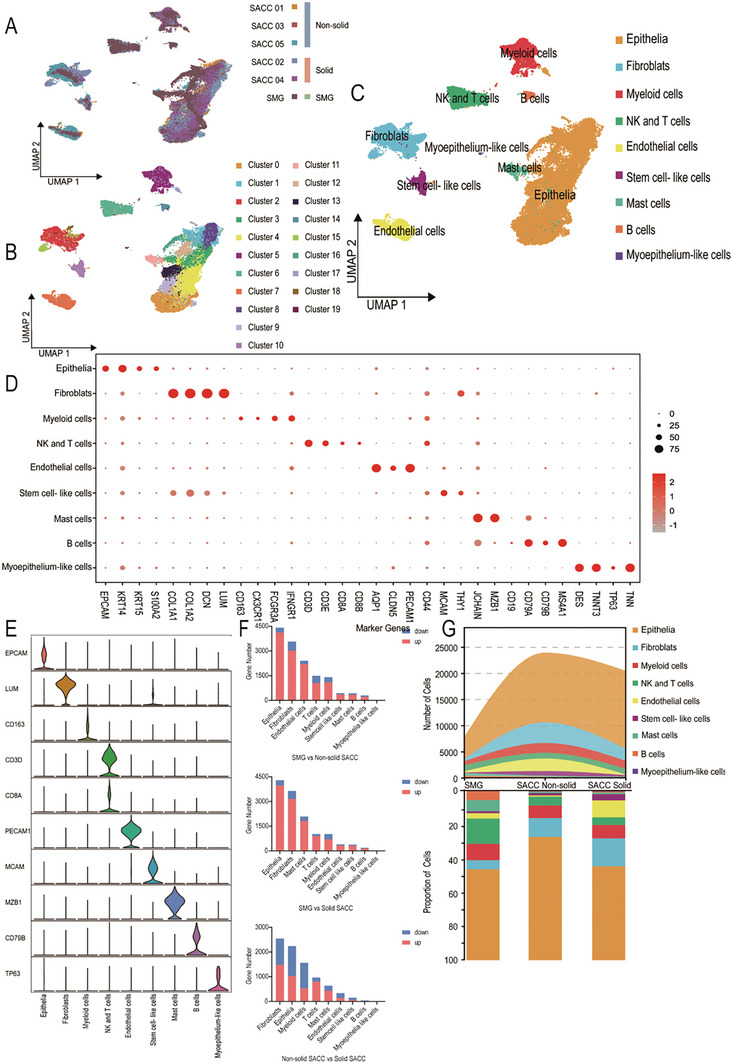
The infiltration of immune cells in SACC is comparatively diminished, particularly in solid‐type SACC. A) Uniform manifold approximation and projection (UMAP) analysis was performed on scRNA‐seq data from both SMG and SACC, with cells labeled by patient sample and histological type. The analysis included 23923 non‐solid SACC cells, 20469 solid SACC cells, and 7826 SMG cells. B) UMAP of scRNA‐seq cells recovered from both SMG and SACC labeled by cluster. Twenty clusters were identified after data combination and batch‐effect correction. C) UMAP of scRNA‐seq cells recovered from both SMG and SACC cells labeled by cell type. Twenty clusters were annotated with nine cell populations. D) Dot plot showing marker gene distributions across nine cell populations. E) Violin plots showing smoothed expression distribution of marker genes in nine cell populations. F) The number of differentially expressed genes (DEG) between groups within the same cell type. The quantification of differentially expressed genes within the same cell populations across distinct histological groups. G) The cell numbers and percentage frequencies of cell populations in scRNA‐seq data among different groups.

To comprehensively investigate the cellular populations of SACC and SMG, we conducted scRNA‐seq on five SACC tumor tissues with diverse histopathological types and a tumor‐matched SMG obtained for surgical resection (**Figure** **1**A). Among the five patients with SACC included in this study, three exhibited a mixed tubular and cribriform pattern, referred to as non‐solid SACC, whereas two presented with a solid component exceeding 30% and were designated as solid SACC. After filtering out the low‐quality cells,^[^ ^18^ ^]^ 23923 non‐solid SACC cells, 20469 solid SACC cells, and 7826 SMG cells were retained for subsequent analyses. To infer cell type identities, unsupervised clustering of 52218 quality control‐passed cells identified 20 clusters after data combination and batch effect correction (Figure 1B).^[^ ^19^ ^]^ These clusters were annotated with nine cell populations, including epithelium, fibroblasts, myeloid cells, T and NK cells, endothelial cells, tissue stem cells, mast cells, B cells, and myoepithelioma‐like cells, using a Single R automated annotation^[^ ^20^ ^]^ combined with manual identification (Figure 1C). The cell populations were characterized based on the expression of specific marker genes, such as EPCAM, KRT15, and KRT14, which are indicative of epithelial cells (Figure 1D,E).^[^ ^12^, ^21^, ^22^, ^23^ ^]^

In addition to the marker genes, the correlation between each cluster and Kyoto Encyclopedia of Genes and Genomes (KEGG) enriched signaling pathways provided further support for our accurate nomenclature. For example, Cluster 5 was defined as myeloid cells enriched in antigen processing and was presented in the KEGG enrichment analysis. Cluster 6 was identified as T and NK cells enriched in T cell receptor signaling pathways and T cell differentiation‐related pathways in the KEGG enrichment analysis, while epithelial‐related clusters were enriched in adhesion junctions, various cancer signaling pathways, and salivary gland secretion (Figures S1–S3, Supporting Information).

To investigate the differential gene expression within the same cell population across SACC types and to provide a novel analytical perspective for understanding the relationship between mRNA changes and diverse phenotypes, we conducted a comparative analysis of the distributed populations. Consequently, we identified genes that exhibited significant differences between pathological subtypes of the same cell population. Our analysis revealed that epithelial cells and fibroblasts were the primary sources of DEG across diverse histopathological classifications (Figure 1F). Therefore, our subsequent experimental studies focused on investigating the characteristics of epithelial cells.

SACC exhibited cellular heterogeneity, with a diverse composition of cells that varied between the different groups (Figure 1G), reflecting the heterogeneity among the different pathological types of SACC. This observation underscores the intratumoral heterogeneity inherent in various SACC pathological subtypes and provides a comprehensive tumor microenvironment landscape for SACC characterization. We found that the infiltration of immune cells, such as NK and T cells, in SACC was comparatively diminished, particularly in solid‐type SACC (Figure 1G). Subsequently, we employed unsupervised clustering analysis to reduce the dimensions of NK and T cells and assigned them distinct names such as NK cells, helper T cells, regulatory T cells, and effector CD8+ cells, based on cell‐specific marker genes (Figure S4A, Supporting Information). By evaluating the proportion of NK and T cell subclusters, we observed a decreased proportion of effector T cells, along with an increased abundance of exhausted T cells and regulatory T cells within the tumor samples (Figure S4B, Supporting Information). Furthermore, solid SACC exhibited a higher proportion of regulatory T cells with inhibitory function and a lower proportion of effector CD8 + lymphocytes than non‐solid SACC (Figure S4B, Supporting Information). This disparity may contribute to the unfavorable prognosis associated with solid SACC cases. Consequently, our findings suggest that immune response suppression occurs in tumors, and that enhancing immune responses is significant for SACC treatment.

### 2.2 Immune Function of SACC is Impaired at Bulk and Single‐Cell Levels

**Figure 2:**
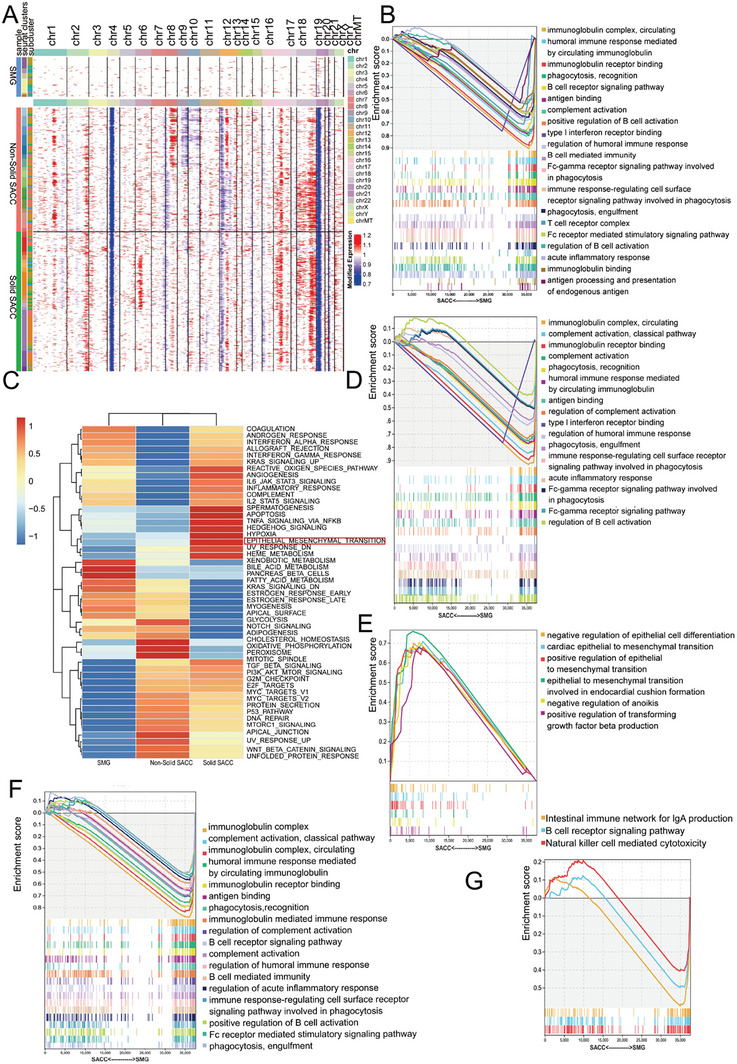
The immune function of SACC is impaired at bulk and single‐cell levels. A) CNV profiles inferred from scRNA‐seq of epithelial cells. Epithelial cells of SMG serve as a reference. B) Representative Gene Set Enrichment Analysis‐Gene Ontology (GSEA‐GO) enrichment pathways in DEG between SMG and SACC at the bulk level (nominal *P‐*value < 0.05, false discovery rate [FDR] < 0.1, sorted by the absolute value of normalized enrichment score (NES)). C) Heatmap showing Gene Set Variation Analysis (GSVA) results exploring the functional roles of different groups of epithelial cells. D,E) Representative GSEA‐GO enrichment pathways in differentially expressed genes of epithelial cells between SMG and SACC at a single‐cell level (nominal *P*‐value < 0.05, FDR < 0.1, sorted by the absolute value of NES). F) Representative GSEA‐GO enrichment pathways in DEG of immune cells between SMG and SACC at a single‐cell level (nominal *P‐*value < 0.05, FDR < 0.1, sorted by the absolute value of NES). G) Representative Gene Set Enrichment Analysis‐Kyoto Encyclopedia of Genes and Genomes (GSEA‐KEGG) enrichment pathways in differentially expressed genes of immune cells between SMG and SACC at a single‐cell level (nominal *P*‐value < 0.05, FDR < 0.1, sorted by the absolute value of NES).

To determine the differences in single‐cell genomics, we performed a Copy Number Variation (CNV) analysis of the SACC epithelia. We found 505 CNV in solid SACC and 503 CNV in non‐solid SACC (**Figure** **2**A; and Table S1, Supporting Information). There was little difference in deletions between solid and non‐solid SACC, with most deletions concentrated on chromosomes 4, 20, and 21. However, significant differences were observed in the CNV amplification. The amplification of MYB, which is essential for the occurrence and progression of SACC, was detected on chromosome 6 in non‐solid ACC, without notable changes in solid SACC. This contrasts with the findings of Xu et al.,^[^ ^24^ ^]^ who indicated that changes in MYB significantly contribute to the pathogenesis of SACC. In this study, the CNV of MYB appeared to have a minimal impact on MYB gene function. The CNV alterations of Notch1–3 on chromosome 19 were observed in solid SACC; both deletion and amplification of Notch1 occurred, whereas only amplification of Notch2 and Notch3 occurred. Notch is considered a key factor in cell proliferation and survival in SACC.^[^ ^25^, ^26^ ^]^

To further investigate the occurrence and developmental mechanisms of SACC, we conducted a comprehensive exploration of the significant DEGs between SMG and SACC at both the bulk and single‐cell levels. Utilizing the DEGs between SMG and SACC at the bulk level, we performed Gene Set Enrichment Analysis (GSEA) to identify diverse biological processes and function‐related pathways between the two. The GSEA revealed that SMG exhibited an enrichment of pathways associated with both innate and acquired immunity, which were suppressed in SACC, including immunoglobulin receptor binding, phagocytosis, type I interferon receptor binding, B cell‐mediated immunity, and acute inflammatory response (Figure 2B). Subsequently, GSVA analysis was performed at the single‐cell level in epithelial cells, which revealed that solid epithelial cells had a higher degree of EMT compared to non‐solid epithelial cells (Figure 2C). Since EMT is a crucial event in cancer invasion and metastasis,^[^ ^27^ ^]^ we postulated that EMT may also play a significant role in SACC, especially solid SACC. This finding is consistent with the observation of enhanced metastatic characteristics within the solid SACC subtype.^[^ ^28^ ^]^ Furthermore, we performed GSEA of epithelial and immune‐related cells at the single‐cell level to elucidate their functions. The GSEA of epithelial cells revealed that pathways related to innate and acquired immunity were enriched in SMG epithelial cells (Figure 2D). The EMT‐related pathways, including negative regulation of epithelial cell differentiation and positive regulation of EMT, were enriched in SACC epithelial cells. Additionally, negative regulation of anoikis and positive regulation of transforming growth factor beta production were enriched in the SACC epithelial cells (Figure 2E). In the GSEA of immune‐related cells, including myeloid cells, mast cells, B cells, NK cells, and T cells, pathways related to both innate and acquired immunity were significantly activated within the immune‐related cells of the SMG (Figure 2F,G). This suggests that the innate and acquired immunity of epithelial cells and immune‐related cells are suppressed in SACC. In summary, it can be inferred that SACC exhibits immunosuppression and EMT, with the EMT more pronounced in solid SACC.

### 2.3 Hybrid Epithelial‐Mesenchymal Transtition (EMT) State Cells Reside in the Vascular‐Fibroblast Microenvironment and are Pro‐Tumorigenic

**Figure 3:**
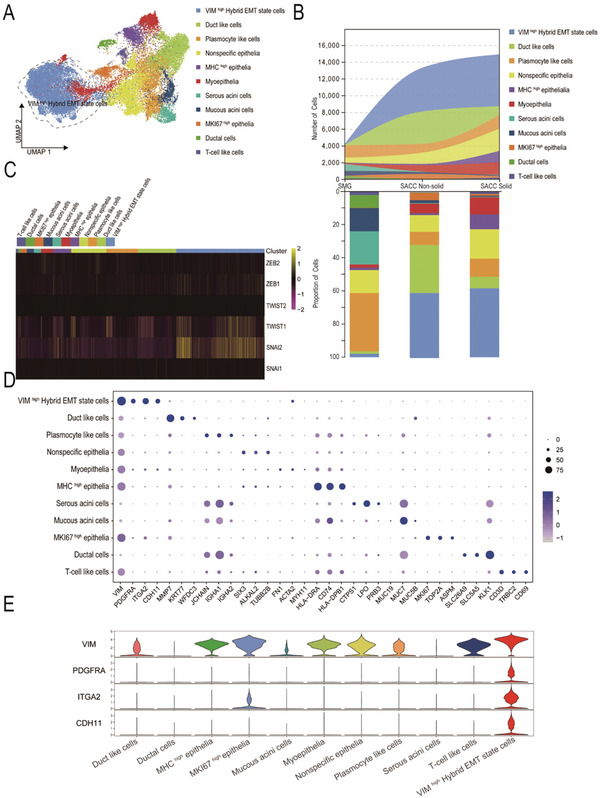
Hybrid EMT cells are characterized by the upregulation of the gene expressions of PDGFRA, ITGA2, and CDH11. A) TheUMAP of epithelial subpopulations from both SMG and SACC labeled by cell type. The cell populations enclosed by dotted lines are hybrid EMT state cells. B) The cell number and percentage frequency of epithelial subpopulations in the scRNA‐seq data among different groups. C) The heatmap displays the expression levels of classical transcription factors that regulate EMT across different epithelial subpopulations. ZEB1, TWIST1, and SNAI1 have specific expression levels within the hybrid EMT state cell population. D) Dot plot showing marker gene distributions across the different epithelial subpopulations. E) Violin plot showing the expression of marker genes during hybrid EMT state cells, with PDGFRA, ITGA2, and CDH11 highly expressed in hybrid EMT state cells.

SACC is an epithelial tumor in which the epithelial cells comprise the principal cellular component,^[^ ^29^ ^]^ accounting for 62% of the total cell population. Further exploration of SACC at the single‐cell level will aid in deciphering the biological features that contribute to the propensity of SACC to metastasize and invade, ultimately fostering the development of novel treatment strategies and improving the prognosis. To delineate functional disparities among epithelial subclusters, we further reduced the epithelium in dimension clustering, resulting in 18 distinct Clusters, which were designated as 11 cell types (**Figure** **3**A; Figure S4C and D) based on the specific gene expression profiles of each cell type (Figure 3D). In the SACC epithelium, we identified a cell subpopulation displaying an intermediate state of EMT called the VIM^high^ hybrid EMT, which encompassed Clusters 0, 1, 15, and 16. This subpopulation highly expressed both the mesenchymal marker and the epithelial marker (Figures 1D and 3D,E). Hybrid EMT cells are closely associated with lung metastasis and tumor cell proliferation. As there was a higher proportion of VIM^high^ EMT cells in the solid SACC (Figure 3B), we postulated that the elevated proportion of VIM^high^ hybrid EMT cells could be associated with the propensity for invasion and metastasis to the lungs.

In summary, Clusters 0, 1, 15, and 16 displayed similar overall characteristics and were located within the hybrid EMT. An analysis of the degree of malignancy by copy number alteration revealed a highly malignant state (Figure S4E, Supporting Information), whereas the cell cycle analysis indicated moderate proliferative activity (Figure S4G, Supporting Information). In this study, we observed that among the six classical transcription factors regulating the EMT process, only ZEB1, Snail2, and Twist1 demonstrated notably augmented expression in VIM^high^ hybrid EMT cells compared to other epithelial cells. This suggests that ZEB1, Snail2, and Twist1 play crucial roles as transcriptional regulators in VIM^high^ hybrid EMT cells (Figure 3C). Additionally, we analyzed the high‐abundance regulons in hybrid EMT cells and found that HMGN3, CEBPB, and JUN may play important regulatory roles (Figure S4F, Supporting Information). We also analyzed the expression of specific genes in the hybrid EMT cells and found that PDGFRA, ITGA2, and CDH11 were specifically expressed (Figure 3D,E).

**Figure 4:**
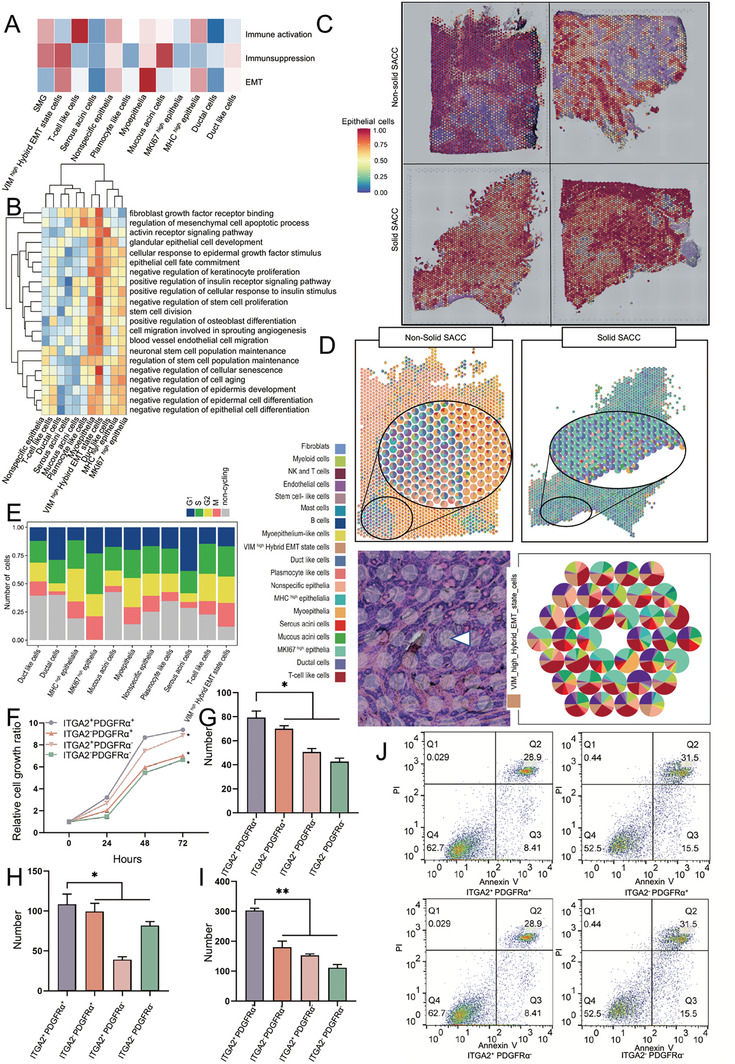
Hybrid EMT state cells reside in the vascular‐fibrous microenvironment and exhibit a pro‐tumorigenic impact. A) Enrichment scores of gene sets linked to immunoactivation, immunosuppression, and EMT across distinct epithelial subpopulations. B) The heatmap showing GSVA across different epithelial subpopulations. C) The spatial distribution score of epithelial cells in the four tumor samples: a higher score indicates a greater likelihood that the region is composed of epithelial cells. D) The top illustration depicts the composition and proportion of ST sequencing cell types and epithelial subsets. The bottom illustration demonstrates the composition and proportion of epithelial subsets surrounding the nerve invaded by the tumor, with a white triangle indicating the location of the nerve. E) The cell cycle analysis shows the percentage of cells in different cell cycle states of epithelial subpopulations. F) Hybrid EMT cells sorted using flow cytometry and cultured for 0, 24, 48, and 72 h. The cell proliferation was assessed with the CCK‐8 assay to calculate the relative rate. Mean ± standard error of the mean (SEM) is shown, *P < 0.05 using one‐way analysis of variance (ANOVA). G) Results of the clone formation experiment, which documented colonies consisting of a minimum of 50 cells. Mean ± SEM is shown, *P < 0.05 using one‐way ANOVA. H,I) Transwell assays performed to assess the cell migration and invasion: the number increases during the migration and invasion of hybrid EMT cells. Mean ± SEM is shown, *P < 0.05 using one‐way ANOVA. J) Hybrid EMT cells sorted using flow cytometry after they were cultured in low‐adhesion culture dishes, harvested after 48 h, and labeled with Annexin V‐FITC and Propidium Iodide (PI).

Subsequently, GSVA was applied to the 11 epithelial cell subsets. Within the hallmark gene sets, the EMT scores of the VIM^high^ hybrid EMT exceeded those of most epithelial cells; however, were lower than those of myoepithelial cells (**Figure** **4**A). Previous studies demonstrated that myoepithelial cells exhibit a predilection for mesenchymal cells, distinguishing them from unique cells within gland tumors. For the first time at the single‐cell level, we identified a subset of epithelial cells characterized by EMT, which is crucial for SACC invasion and metastasis. In addition, we performed GSEA on epithelial cells and observed that VIM^high^ hybrid EMT cells exhibited a higher propensity for mesenchymal characteristics, but negatively regulated epithelial cell differentiation and development (Figure 4B).

To delineate the spatial localization of VIM^high^ hybrid EMT cells, we conducted spatial transcriptome analysis of the four samples. Our findings revealed that epithelial cells constituted the predominant cellular component of the tumor, which was consistent with previous single‐cell data (Figure 4C). Spatial localization revealed that VIM^high^ hybrid EMT cells colocalized with endothelial cells and fibroblasts, suggesting their presence within a microenvironment characterized by vascular fibrosis (Figure 4D). Furthermore, we observed that VIM^high^ hybrid EMT cells were located around the invaded nerves, indicating their potential involvement in tumor invasion into the nerves (Figure 4D). Additionally, we conducted a cell cycle analysis, which indicated a high level of proliferative activity in the hybrid EMT cells (Figure 4E).

To isolate VIM^high^ hybrid EMT cells for in vitro functional studies, we sorted epithelial cells using specific surface markers called PDGFRα and ITGA2. We observed that PDGFRα+ and ITGA2+ cells demonstrated enhanced colony formation, migration, invasion, proliferation, and anoikis resistance compared with others (Figure 4F–J; Figure S2F–I, Supporting Information). This further confirmed that the hybrid EMT cells were pro‐tumorigenic. Consequently, a targeted hybrid EMT cell therapy holds promise for delaying SACC progression and mitigating lung metastasis.

### 2.4 Cadherin 11 (CDH11) Modulates the Function of Hybrid EMT Cells

**Figure 5:**
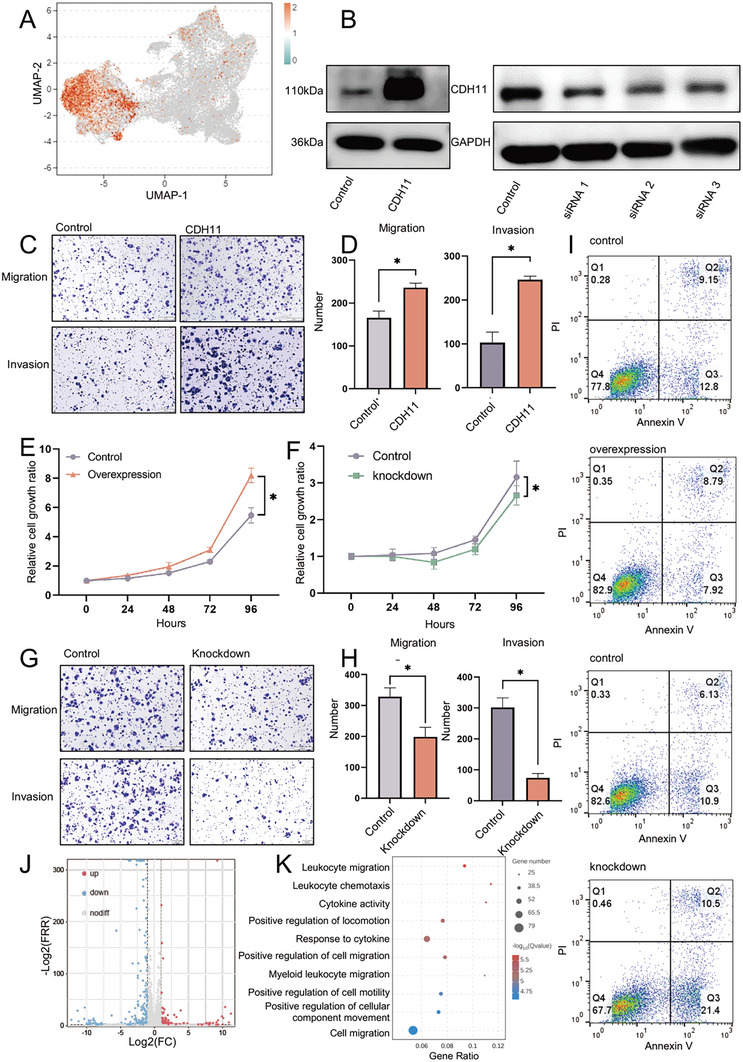
CDH11 acts as a biomarker for hybrid EMT state cells, playing a crucial role in promoting cell proliferation, migration, invasion, and anoikis resistance. A) The UMAP shows the expression distribution of CDH11 in epithelial cells, and CDH11 is highly expressed in hybrid EMT state cells. B) Efficiency of CDH11 overexpressed or knocked down by a plasmid or small interfering RNA (siRNA) in SACC‐83 cells measured on western blotting. C,D) CDH11‐overexpressed SACC‐83 cells analyzed for their migration and invasion ability using transwell assays. The number of migrated and invaded cells was counted (*n *= 3, *P < 0.05). Scale bar, 200 µm, Mean ± SEM is shown, *P < 0.05 using *t* test. E,F) CDH11‐overexpressed or ‐knockdown SACC‐83 cells cultured for 0, 24, 48, and 72 h, and cell proliferation determined using the CCK‐8 assay. Mean ± SEM is shown, *P < 0.05 using *t* test. G,H) CDH11‐knockdown SACC‐83 cells analyzed for their migration and invasion ability using transwell assays. The number of migrated and invaded cells was counted (*n *= 3, *P < 0.05). Scale bar, 200 µm, Mean ± SEM is shown, *P < 0.05 using *t* test. I) CDH11‐overexpressed or ‐knockdown SACC‐83 cells cultured in low‐adhesion culture dishes, harvested after 48 h, and labeled with Annexin V‐FITC and PI were subjected to flow cytometry. J) Volcano plot showing differentially expressed genes in SACC‐83 cells after CDH11 overexpression, as identified via RNA‐seq. K) Top 10 terms of GO enrichment pathways in SACC‐83 cells after CDH11 overexpression.

To identify the therapeutic targets for hybrid EMT cells, we screened for marker molecules specifically expressed in hybrid EMT cells and found that cadherin 11 (CDH11) was highly expressed (**Figure** **5**A; Figure S4A, Supporting Information). To elucidate the relationship between CDH11 expression and prognosis in SACC, we assessed the mRNA levels of CDH11 in 120 patients. Our findings indicate that higher levels of CDH11 are associated with poorer outcomes, whereas lower expression correlates with a more favorable prognosis in SACC (Figure S4B, Supporting Information). Therefore, further investigations into the role of CDH11 may enhance our understanding of its mechanism of action in SACC and ultimately improve the prognosis of patients with SACC. Then, we assessed the suitability of CDH11 as a potential therapeutic target for SACC and observed its robust expression in SACC, specifically in hybrid EMT cells and cancer‐associated fibroblasts (CAFs) (Figure S4C–E, Supporting Information). The CAFs have been implicated in tumor development,^[^ ^30^ ^]^ and CDH11 may be a promising therapeutic target for both hybrid EMT cells and CAFs. To validate the functional role of CDH11 in SACC, we performed CDH11 knockdown, resulting in the inhibition of proliferation, resistance to anoikis, and migration and invasion of SACC‐83 cells. However, the overexpression of CDH11 in SACC‐83 cells led to increased proliferation, migration, and invasion (Figure 5B–I). The RNA‐seq of CDH11‐overexpressing SACC‐83 cells revealed enrichment of the positive regulation of cell migration in Gene Ontology (GO) analysis (Figure 5J,K).

**Figure 6:**
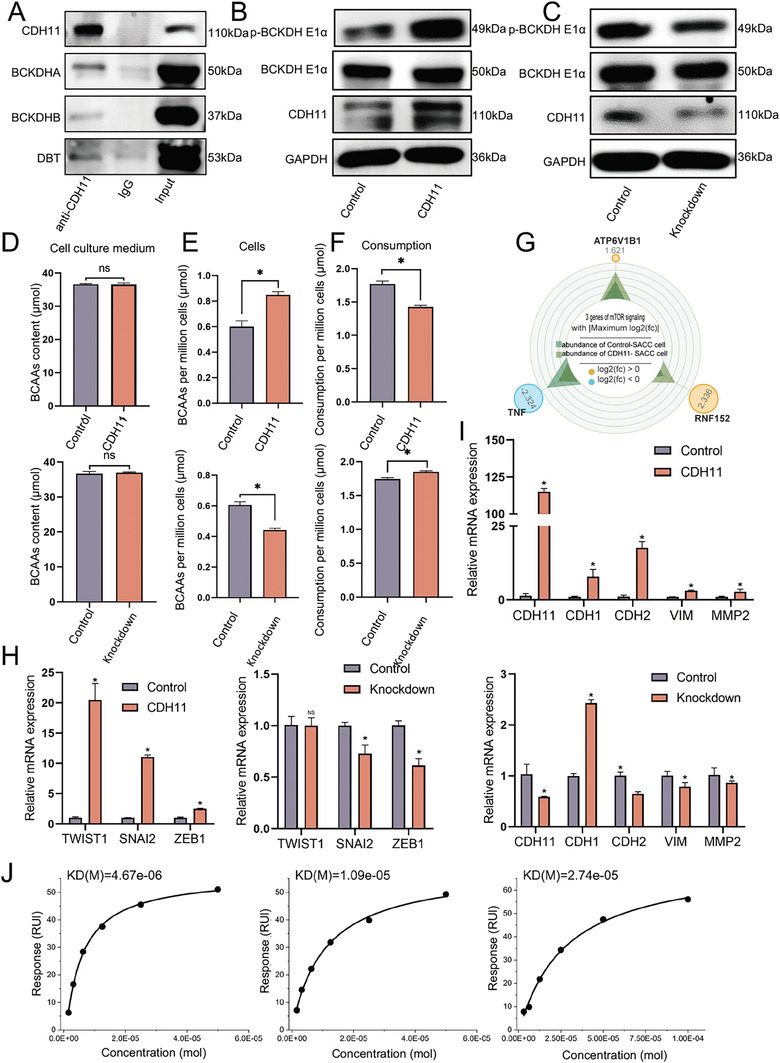
CDH11 disrupts the catabolism of BCAA leading to the activation of the mechanistic target of rapamycin pathway. A) The co‐immunoprecipitation assay demonstrates the interaction between CDH11 and BCKDHA, BCKDHB, and DBT proteins. B,C) Western blot showing increased phosphorylation levels of BCKDHE1α in CDH11‐overexpressed SACC‐83 cells. The results are reversed in CDH11‐knockdown cells. D) BCAA content in the culture medium shows no significant change after culturing SACC‐83 cells with CDH11 overexpression or knockdown for 24 h. *n *= 3, Mean ± SEM is shown. *P < 0.05 using *t* test. E) BCAA content per million SACC‐83 cells exhibits an increase following 24 h of CDH11‐overexpressed cell culture. The results are reversed in CDH11‐knockdown cells. *n *= 3, Mean ± SEM is shown, *P < 0.05 using *t* test. F) BCAA consumption per million SACC‐83 cells exhibits a decrease after culturing 24 h in CDH11‐overexpressed cells. The results are reversed in CDH11‐knockdown cells. *n *= 3, Mean ± SEM is shown, *P < 0.05 using *t* test. G) RNA‐seq analysis shows an upregulation of ATP6V1B1 and rnf152 gene expression and a downregulation of TNF gene expression in SACC‐83 cells with CDH11 overexpression. H) Quantitative reverse transcription polymerase chain reaction (qRT‐PCR) analysis shows that overexpressing CDH11 in cells significantly increased the expression of EMT‐associated transcription factors. The results are reversed in CDH11‐knockdown cells. *n *= 3, Mean ± SEM is shown, *P < 0.05 using *t* test. I) qRT‐PCR analysis reveals a significant upregulation of EMT‐associated genes in cells overexpressing CDH11. The results are reversed in CDH11‐knockdown cells. *n *= 3, Mean ± SEM is shown, *P < 0.05 using *t* test. J) The Dissociation Constants (KD) of Celecoxib (CXB), Dimethylcelecoxib (DMC) and SD‐133 with the CDH11 protein detected by Surface plasmon resonance were 4.67e‐06, 1.09e‐05, and 2.74e‐05 M, respectively.

In addition to its role as an effector molecule, CDH11 exerts regulatory effects on tumor cell function through alternative signaling pathways. We employed a co‐immunoprecipitation assay coupled with mass spectrometry to identify potential interacting molecules of CDH11 (Figure S3E,F, Supporting Information). Our findings reveal a novel interaction between CDH11 and the branched‐chain ketoacid dehydrogenase (BCKDH) complex (**Figure** **6**A; Figure S4F,G, Supporting Information). The BCKDH complex is a rate‐limiting enzyme in branched‐chain amino acid (BCAA) metabolism and comprises three subunits: BCKDHA, BCKDHB, and DBT. This complex is activated or inactivated by the dephosphorylation or phosphorylation of BCKDHA, respectively.^[^ ^31^ ^]^ The BCAAs are metabolized by a cascade of enzymes into α‐keto acids, which are ultimately catabolized by BCKDH to generate acetyl‐CoA and succinyl‐CoA (R‐CoA), that enter the tricarboxylic acid cycle (TAC).^[^ ^31^ ^]^ Our findings suggested that CDH11 plays a regulatory role in BCAA catabolism.

The phosphorylation of BCKDHA was elevated in CDH11‐overexpressed SACC‐83 cells, indicating that BCKDHA was inactive (Figure 6B,C). Subsequently, the metabolism of BCAAs was assessed after 24 h of culture using a BCAA detection kit. We observed no significant change in the extracellular concentration of BCAAs after 24 h, however, there was a decrease in BCAA consumption per million cells and a increase in intracellular BCAA storage per million cells in CDH11‐overexpressed SACC‐83 cells compared to the control group after 24 h. Conversely, CDH11‐knockdown SACC‐83 cells exhibited an inverse trend. This suggests that CDH11 does not affect the cellular uptake of BCAAs but rather influences their catabolism by binding to the BCKDH complex, ultimately leading to reduced R‐CoA and increased intracellular BCAAs, which exert regulatory effects on cell functions through alternative pathways (Figure 6D–F). Previous studies have reported the potential impact of BCAAs on the proliferation, migration, and invasion of tumor cells via the mammalian target of the rapamycin (mTOR) pathway.^[^ ^32^, ^33^ ^]^ Through the RNA‐seq of SACC‐83 cells overexpressing CDH11, we observed a significant upregulation of RNF152 and ATP6V1B1, which are responsible for sensing BCAAs and regulating mTOR complex 1 in the mTOR pathway (Figure 6G). Conversely, downregulation of TNF was observed (Figure 6G). mTOR activation enhances the expression of EMT‐related transcription factors. The transcription factors TWIST1, SNAI2, and ZEB1 were found to exhibit high expression levels in CDH11‐overexpressing cells, which regulated hybrid EMT cells, accompanied by the upregulation of EMT‐related mRNA. In contrast, the knockdown of CDH11 resulted in downregulation of the transcription factors SNAI2 and ZEB1, along with decreased expression levels of EMT‐related mRNA (Figure 6H,I). Therefore, we propose that CDH11 disrupts BCAA metabolism through BCKDH, hindering BCAA catabolism and the subsequent activation of the mTOR pathway. Ultimately, this regulatory cascade modulates EMT via SNAI2 and ZEB1. However, a comprehensive understanding of the precise mechanisms underlying SACC remains elusive and warrants further investigation.

### 2.5 Celecoxib (CXB) Inhibits Lung Metastasis of SACC by Targeting CDH11 and Activating the Cyclic GMP‐AMP Synthase‐Stimulator of Interferon Genes Pathway through Reactive Oxygen Species (ROS)

**Figure 7:**
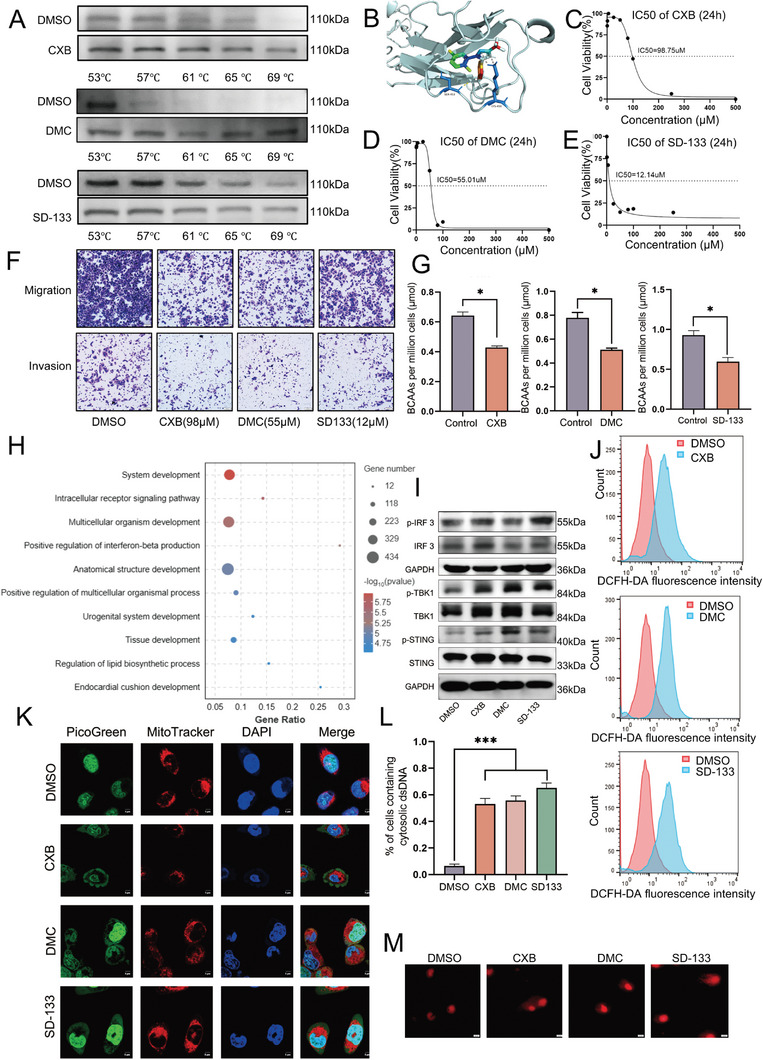
Combination of CXB/DMC/SD‐133 with CDH11 activates the cyclic GMP‐AMP synthase‐stimulator of the interferon gene (cGAS‐STING) pathway through ROS, suppressing cellular invasion and migration. A) Representative western blots of the cellular thermal shift assay showing increase in CDH11 thermostable performance in the presence of CXB, DMC, and SD‐133. B) SD‐133 with interactive residue side chains at the pocket are shown in stick rendering, with the inhibitors drawn in colorful. The polypeptide backbones are rendered as ribbons. The yellow broken lines indicate potential intermolecular hydrogen bonds, while the gray broken lines indicate pi‐cation interactions. C–E) Effect of CXB, DMC, and SD‐133 on the inhibition of SACC‐83 cells; normalized data and non‐linear regression curve fitting are shown. IC_50_ values are indicated. F) BCAA per million SACC‐83 cells exhibited a decrease 24 h after treatment with CXB, DMC, or SD‐133. Mean ± SEM is shown, *P < 0.05 using *t* test. G) SACC‐83 cells treated with CXB, DMC, or SD‐133 analyzed for their migration and invasion ability using transwell assays. Scale bar, 100 µm, *n *= 3, Mean ± SEM is shown, *P < 0.05 using *t* test. H) Top 10 terms of GO enrichment analysis of DEG by RNA‐seq in SACC‐83 cells treated with SD133. I) Expression of cGAS‐STING pathway‐related proteins in SACC‐83 cells, treated with CXB, DMC, and SD 133, assessed using western blotting. J) Flow cytometry reveals an increase in reactive oxygen species (ROS) production after treatment with CXB, DMC, or SD‐133. K,L) Confocal microscopy showing the accumulation and quantification of cytosolic deoxyribonucleic acid (DNA) in SACC‐83 cells following treatment with CXB, DMC, or SD‐133. Double‐stranded DNA (dsDNA) visualized using PicoGreen staining (green), while MitoTracker (red) and DAPI (blue) employed to label mitochondria and nuclei, respectively. Scale bar, 5 µm. More than 100 cells were analyzed per group. *n *= 10, Means ± SEM is shown. ***P < 0.001 using one‐way analysis of variance. M) Representative images of DNA comet assays of SACC‐83 cells subjected to treatment with CXB, DMC, or SD‐133.

We conducted a comprehensive literature review to identify the targeted therapeutic drugs for CDH11. A previous study demonstrated the potential of CXB, a non‐steroidal anti‐inflammatory drug, to bind and inhibit CDH11, thereby effectively suppressing the cell invasion and migration.^[^ ^34^, ^35^, ^36^ ^]^ However, it is important to note that CXB is primarily utilized as an anti‐Cyclooxygenase‐2 (COX‐2) agent in cancer treatment. Thus, further investigation is required to determine its efficacy in inhibiting CDH11 for tumor treatment. In contrast, DMC, a derivative of CXB, specifically targets and inhibits CDH11 without affecting COX‐2 activity.^[^ ^37^ ^]^ Another CXB derivative, SD‐133, was identified as a binding inhibitor for CDH11.^[^ ^36^ ^]^ To evaluate the binding affinity of these compounds for CDH11, we conducted a cellular thermal shift assay using SACC‐83 cells treated with median inhibitory concentration (IC_50_) concentrations. All three compounds exhibited significant binding capability toward CDH11 (**Figure** **7**A; Figure S5H, Supporting Information). Surface plasmon resonance (SPR) was used to further validate the binding affinity between the drugs and CDH11. The KD of CXB, DMC, and SD‐133 interacting with the CDH11 protein were 4.67e‐06, 1.09e‐05, and 2.74e‐05 M, respectively (Figure 6J; Figure S6A, Supporting Information). Notably, all the three drugs exhibited strong binding affinities for CDH11. Additionally, a molecular docking analysis of the protein‐small‐molecule drug demonstrated that the three drugs exhibited a strong structural stability within the active pocket region of the protein, binding to CDH11 through covalent and hydrogen bonds (Figure 7B; Figure S6B,C, Supporting Information). In summary, these experiments provided evidence supporting the binding of celecoxib and its derivatives to CDH11. The IC_50_ values of CXB, DMC, and SD‐133 were 98.75, 55.01, and 12.14 µm, respectively (Figure 7C–E). Furthermore, these drugs induced apoptosis in tumor cells and effectively suppressed the tumor cell invasion and migration (Figure 7F); cellular BCAA were significantly reduced after drug treatment (Figure 7G). Subsequently, RNA‐seq was performed on cells treated with SD133. The GO analysis revealed that SD133 treatment led to the enrichment of innate immunity and type I interferon production pathways (Figure 7H). The cGAS‐STING pathway functions as a direct upstream regulatory factor that governs type I interferon production.^[^ ^38^ ^]^ Therefore, we investigated the effect of drug treatment on the cGAS‐STING pathway activation and found that it was activated by CXB, DMC, and SD‐133 (Figure 7I).

After treatment with CXB, DMC, or SD‐133, the cellular BCAA content decreased, thereby substantiating the partial correction of BCAA metabolic disorders, and facilitating the normal entry of metabolites into the TAC. Consequently, we postulated a potential association between the activation of the cGAS‐STING pathway and ROS generation during metabolism. The involvement of BCAA metabolites in TAC metabolism has been extensively studied. Therefore, our investigation focused on elucidating the generation of ROS within the TAC because its elevation in these species directly contributes to the production of free DNA fragments. Flow cytometry analysis revealed that treatment with CXB, DMC, or SD133 enhanced the ROS generation (Figure 7J). After staining cytoplasmic‐free dsDNA with PicoGreen, a significant increase was observed following treatment with CXB, DMC, and SD‐133 compared to that in the untreated group (Figure 7K,L). Comet assays also revealed that the drug treatment significantly increased the comet tail DNA compared to that in the untreated group (Figure 7M; Figure S5I, Supporting Information). Therefore, we proposed that CDH11 regulates BCAA metabolism to modulate ROS generation, thereby regulating the cGAS‐STING pathway. The administration of CXB, DMC, or SD‐133 can cause BCAAs to undergo normal metabolism into TAC, resulting in elevated ROS production and cytoplasmic free DNA generation, which trigger the activation of the cGAS‐STING pathway.

**Figure 8:**
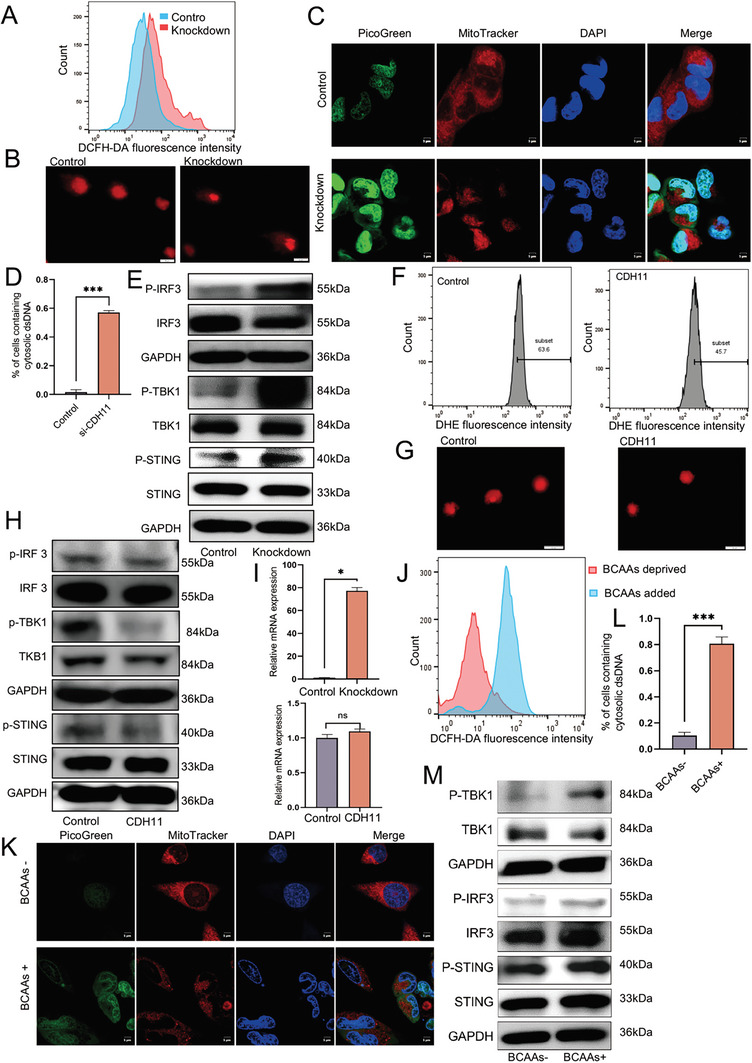
Metabolism of BCAA increases the production of ROS, activating the cGAS‐STING pathway. A) Flow cytometry detected ROS production in SACC‐83 cells with CDH11 knockdown. B) Representative images of DNA comet assays of SACC‐83 cells subjected to various experimental conditions. Scale bar, 20 µm. C,D) Confocal microscopy showing the accumulation and quantification of cytosolic DNA in SACC‐83 cells under knockdown CDH11. dsDNA visualized using PicoGreen staining (green), while MitoTracker (red) and DAPI (blue) employed to label mitochondria and nuclei, respectively. Scale bar, 5 µm. More than 100 cells were analyzed per group. Mean ± SEM is shown. *n *= 10, ***P < 0.001 using *t* test. E) Expression of cGAS‐STING pathway‐related proteins in SACC‐83 cells, treated under various experimental conditions, assessed using western blotting. F) Flow cytometry of SACC‐83 cells overexpressing CDH11 to evaluate the mitochondrial activity. G) Representative images of DNA comet assays of SACC‐83 cells subjected to various experimental conditions. Scale bar, 20 µm. H) Expression of cGAS‐STING pathway‐related proteins in SACC‐83 cells, treated under various experimental conditions, assessed using western blotting. I) qRT‐PCR analysis shows that CDH11‐knockdown cells significantly increase the expression of IFNB1. No significant difference in the overexpression group. *n *= 3, Mean ± SEM is shown, *P < 0.05 using *t* test. J) Flow cytometry detected ROS production in BCAA‐ deprived or ‐added SACC‐83 cells. K,L) Confocal microscopy showing the accumulation and quantification of cytosolic DNA in BCAA‐deprived or ‐added SACC‐83 cells. dsDNA visualized using PicoGreen staining (green), while MitoTracker (red) and DAPI (blue) employed to label mitochondria and nuclei, respectively. Scale bar, 5 µm. More than 100 cells were analyzed per group. *n *= 10, Mean ± SEM is shown. ***P < 0.001 using one‐way analysis of variance. M) The expression of cGAS‐STING pathway‐related proteins in SACC‐83 cells, treated under various experimental conditions, was assessed using western blotting.

Based on these experimental results, we propose that CXB, DMC, and SD‐133 possess the potential to activate the cGAS‐STING pathway through the inhibition of CDH11. Furthermore, our results suggested a plausible association between CDH11 and cGAS‐STING pathway. The ROS production and activation of the cGAS‐STING pathway were investigated in SACC‐83 cells with CDH11 knockdown or overexpression. Flow cytometry revealed that CDH11 knockdown upregulated the ROS production in SACC cells, whereas CDH11 overexpression decreased the mitochondrial activity (**Figure** **8**A,F). In CDH11‐knockdown cells, enhanced cytoplasmic dsDNA staining, increased comet tail DNA, and activation of the cGAS‐STING pathway were observed, resulting in increased downstream INFB1 production (Figure 8B–E,I; Figure S5I, Supporting Information). In contrast, the comet assay performed for SACC cells overexpressing CDH11 showed no noticeable changes in the cytoplasmic dsDNA; however, displayed an inhibition of the cGAS‐STING pathway, resulting in a decrease in downstream INFB1 production (Figure 8G–I; Figure S5I, Supporting Information). In the context of the normal metabolic pathway of BCAA, a reduction in cellular ROS production and dsDNA levels was observed upon culturing in BCAA‐deprived medium, resulting in the inhibition of the cGAS‐STING pathway (Figure 8J–M; Figure S5I, Supporting Information).

Therefore, we proposed that CDH11 regulates BCAA metabolism to modulate ROS generation, thereby regulating the cGAS‐STING pathway. The knockdown of CDH11 or administration of CXB, DMC, and SD‐133 can cause BCAAs to undergo normal metabolism into TAC, resulting in elevated ROS production and cytoplasmic‐free dsDNA generation, which trigger the activation of the cGAS‐STING pathway.

### 2.6 CXB, Dimethylcelecoxib, or SD‐133 Treatments Inhibited Lung Metastasis of SACC in NOD‐SCID Mice

**Figure 9:**
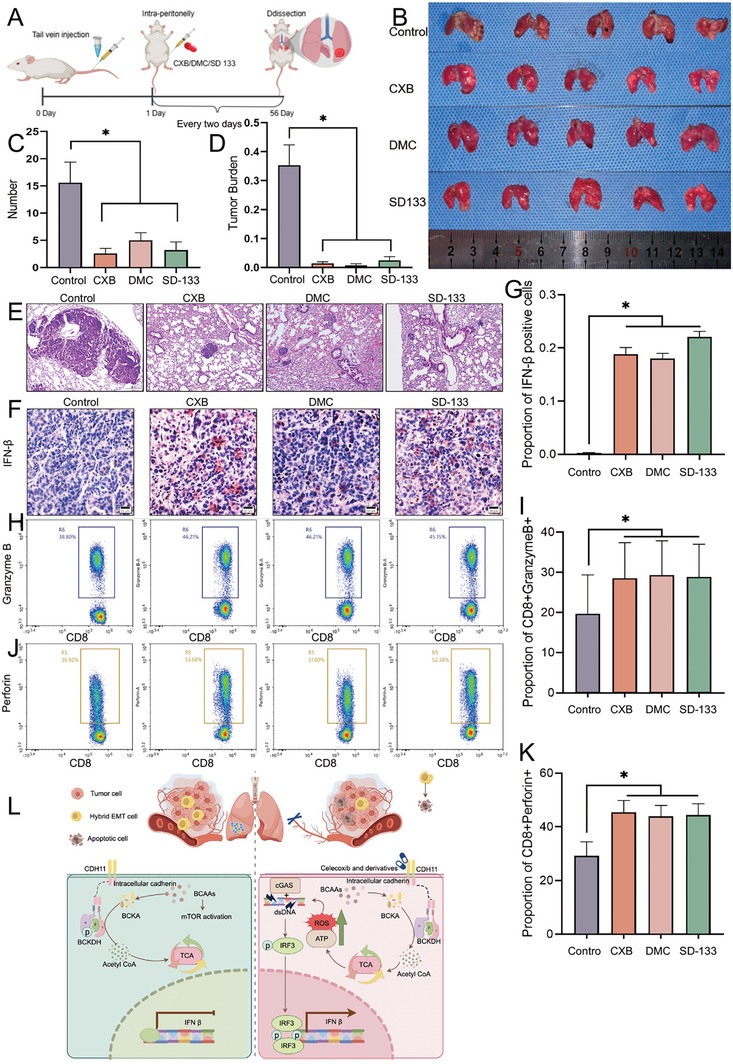
CXB, DMC, or SD‐133 treatment inhibited the lung metastasis of SACC in NOD‐SCID mice. A) Flow diagram of animal research procedures. B) The lungs of NOD‐SCID mice were examined macroscopically. C) Lung metastatic tumor nodules quantified in NOD‐SCID mice. *n *= 5, Mean ± SEM is shown. *P < 0.05 using ANOVA. D) Quantification of the metastatic burden in NOD‐SCID mice. *n *= 5, Mean ± SEM is shown. *P < 0.05 using one‐way ANOVA. E) Lung tissues of NOD‐SCID mice on hematoxylin and eosin staining. Scale bar, 100 µm. F,G) Representative immunohistochemical images and quantification of IFN‐β positive cells in lung metastasis sections from NOD‐SCID mice are presented. Scale bar, 20 µm. *n *= 5, Mean ± SEM is shown. *P < 0.05 using one‐way ANOVA. H,I) The flow analysis diagram showed the change in proportions of Granzyme B+ cytotoxic T cells after drug stimulation. The experiments were performed three times for statistical analysis. *n *= 3, Mean ± SEM is shown. *P < 0.05 using one‐way ANOVA. J,K) The flow analysis diagram showed the change in proportions of Perforin+ cytotoxic T cells after drug stimulation. The experiments were performed three times for statistical analysis. *n *= 3, Mean ± SEM is shown. *P < 0.05 using one‐way ANOVA. L) Schematic diagram elucidating the activation of the cGAS‐STING pathway by celecoxib and its derivatives (By Figdraw, https://www.figdraw.com).

In vivo, NOD‐SCID mice were intravenously injected with SACC‐83 cells to establish a pulmonary metastasis model, and the three drugs were administered concurrently (**Figure** **9**A). After 8 weeks of treatment, the mice were euthanized, and a significant reduction in the number of surface nodules on the lung metastases was observed. Mice treated with CXB, DMC, or SD133 exhibited significantly fewer surface nodules than the control group (Figure 9B,C). Hematoxylin‐eosin staining (H&E) staining of the collected lung tissue also revealed a markedly reduced tumor nodule area in the treated group compared to that in the control group (Figure 9D,E). These results indicate that all the three drugs effectively suppressed the lung metastasis in SACC, although no significant differences were observed. To evaluate the cytotoxicity of the three drugs, H&E staining was performed to specifically target the heart, liver, spleen, and kidneys. The results demonstrated that a normal morphology was observed in both the treatment and control groups (Figure S6D, Supporting Information). Although DMC and SD‐133 did not inhibit COX‐2, they bound to CDH11 and effectively inhibited lung metastasis. Consequently, targeting CDH11 holds a great promise for drug therapy, as it can significantly suppress the tumor lung metastasis. CXB exhibits the dual inhibition of COX‐2 and CDH11, and clinical trials have demonstrated favorable biological safety profiles. Consequently, CXB has emerged as a promising therapeutic option for tumor treatment; however, further investigation is warranted to determine its optimal dosage.

We further employed Iimmunohistochemical staining was employed to examine immune system activation and detect the production of interferon beta (IFN‐β) in lung metastatic tumors of NOD‐SCID mice after drug treatment, as IFN‐β is a downstream product activated following cGAS‐STING activation. We observed a significantly higher number of cells exhibiting positive expression of IFN‐β after treatment compared to the control group (Figure 9E). This finding is consistent with our previous experimental results; inhibition of CDH11 in SACC cell lines also led to the activation of the IFN‐β production pathway. To replicate the antitumor immune microenvironment, monocytes (including mononuclear macrophages and lymphocytes) were isolated from the peripheral blood of healthy individuals and co‐cultured with tumor cells treated with CXB, DMC or SD‐133. The lymphocytes present in the supernatant were collected for analysis. The proportions of CD8+Perforin+ and CD8+Granzyme B+ T cells within the CD8+ population were assessed to monitor changes in cytotoxic T cell proportions after drug treatment (Figure S6E, Supporting Information). Remarkably, the proportion of cytotoxic T cells after co‐culture with treated SACC‐83 cells was significantly higher than that observed in the control group (Figure 9H–K). The experimental results demonstrate that a successful activation of immune responses occurs following CDH11 inhibition‐mediated cGAS‐STING activation, in which IFN‐β plays a crucial role in stimulating tumor‐specific T cell response and may act as a mediator between the CDH11 inhibitor and activation of immune responses.

Collectively, we discovered that CXB and its derivatives possess the ability to enhance ROS production by inhibiting CDH11 and regulating BCAAs metabolism. Consequently, this leads to an increase in the quantity of dsDNA, activates the cGAS‐STING pathway, and effectively suppresses SACC metastasis within the pulmonary region. IFN‐β released after CXB, DMC, or SD133treatment plays a crucial role in stimulating tumor‐specific T cell response and may act as a mediator between the CDH11 inhibitor and immune response activation (Figure 9L).

## 3 Discussion

Currently, surgical treatment of primary tumors significantly reduces the mortality rate of SACC; however, lung metastasis and the lack of effective targeted therapies remain crucial factors influencing the prognosis of patients with SACC.^[^ ^39^, ^40^ ^]^ Therefore, it is essential to explore novel therapeutic strategies for reducing lung metastasis and SACC progression. A comprehensive understanding of the heterogeneity of SACC and elucidation of the underlying molecular mechanisms driving tumor progression are necessary to develop effective treatment strategies. Previous studies have identified potential driver mutations and enriched signaling pathways in SACC through genome and RNA‐seq analyses, thus providing direction for targeted chemotherapy or biological treatment of SACC.^[^ ^1^, ^6^ ^]^

Persson et al. demonstrated that MYB‐NFIB fusion is a distinct characteristic of SACC and that the abnormal regulation of MYB and its downstream target genes resulting from this gene fusion plays a crucial role in the development of SACC.^[^ ^41^ ^]^ However, therapeutic interventions targeting MYB have not shown satisfactory efficacy.^[^ ^42^ ^]^ Additionally, MYB overexpression in the SACC cell line significantly enhances cellular proliferation, migration, and invasion capabilities, accompanied by the upregulation of EMT‐related molecules.^[^ ^24^ ^]^ However, these sequencing methods have limitations. They sequence bulk tumor tissues, resulting in an average value from mixed cells and obscuring specific information about the individual cells within a population, thereby losing cellular heterogeneity. Tumor tissues consist of diverse cell types, including malignant, immune, and stromal cells; however, their precise characteristics are usually concealed using traditional sequencing methods. Additionally, the features associated with tumor recurrence and metastasis often rely on specific cell types that may be obscured by bulk tissue sequencing. The combination of scRNA‐seq and ST enables comprehensive exploration of single‐cell molecular characteristics in SACC, including heterogeneity and spatial distribution.^[^ ^43^, ^44^ ^]^ This integration holds great promise for identifying molecular markers at the single‐cell level and facilitating precise tumor diagnosis, targeted therapy, and prognosis prediction in SACC. Ultimately, this will enhance our understanding of the molecular mechanisms underlying SACC and contribute to the development of personalized tumor treatments.

The study of EMT has revealed a nuanced understanding, indicating that it is not a binary occurrence but rather a continuous and intricate series of events. Pastushenko et al. identified at least seven distinct EMT subsets of cancer cells in skin and breast cancer tissues, each with unique characteristics related to growth, invasion, metastasis, and differentiation.^[^ ^10^, ^11^ ^]^ Notably, the hybrid EMT state plays a significant role in lung metastasis, emphasizing the importance of understanding tumor heterogeneity.^[^ ^10^ ^]^ Furthermore, Liu et al. investigated the specific EMT states of circulating tumor cells crucial for cancer metastasis and suggested that cell subtypes primarily composed of epithelial cells exhibit stronger lung metastasis and proliferation ability. This indicates that hybrid EMT may serve as a superior biomarker of distant metastasis and poor prognosis in patients with breast cancer.^[^ ^45^ ^]^

In this study, the single‐cell profile of SACC was mapped using scRNA‐seq and hybrid EMT cells were identified for the first time in SACC. Using ST sequencing, we found that these cells were localized within the microenvironment of vascularfibroblasts. In vitro experiments revealed that these cells exhibit higher proliferation, migration, invasion, and anoikis resistance than other tumor cells. CDH11 was found to have high expression levels in hybrid EMT cells. Thus, CDH11 is a potential therapeutic target in SACC. Studies have shown that CDH11 is widely expressed during embryonic development, however, its expression is virtually absent in adult cells.^[^ ^46^ ^]^ In tumor tissues, CDH11 is expressed in CAFs and certain epithelial cells, and is associated with the malignant phenotype of tumors.^[^ ^47^, ^48^, ^49^ ^]^ Therefore, a targeted therapy against CDH11 could impede tumor progression without affecting normal cell tissues. CDH11 not only acts as an effector molecule but also influences tumor cell function by interfering with BCAA metabolism and activating the mTOR pathway. CXB, DMC, and SD‐133 effectively inhibited lung metastasis in SACC by targeting CDH11 both in vitro and in vivo. The knockdown of CDH11 or treatment with CXB, DMC, or SD‐133 can lead to normal BCAA metabolism, resulting in the upregulation of ROS production and cytoplasmic‐free dsDNA generation, which subsequently activate the cGAS‐STING pathway. Additionally, cells with high expression of the major histocompatibility complex were observed in epithelial cells, whereas immune cells within the tumor microenvironment were typically suppressed, posing challenges for the effective elimination of tumor cells. Therefore, the successful activation of immune responses within the tumor is crucial for the treatment of SACC. Studies have shown that activation of the STING pathway in tumor cells can induce apoptosis of tumor cell and enhance the immune response to kill tumor cells.^[^ ^50^, ^51^, ^52^ ^]^ The inhibition of CDH11 leads to cGAS‐STING activation, which offers the advantage of tumor specificity. The traditional STING agonist development focuses on cyclic dinucleotides (CDNs), both natural and synthetic CDNs have been used for systemic administration.^[^ ^53^, ^54^ ^]^ However, since STING is expressed in various cell types, including cancer and non‐cancer cells, this class of drugs lacks tumor specificity. Consequently, systemic administration of STING agonists has the potential to indiscriminately activate STING, leading to cell death in both tumor and non‐tumor tissues.^[^ ^55^, ^56^ ^]^ Our study demonstrated that CDH11 was highly expressed in hybrid EMT cells. Therefore, the activation of cGAS‐STING mediated by a CDH11 inhibitor offers specific advantages, as it selectively targets tumor tissue when administered systemically, eliciting an antitumor immune response within the tumor microenvironment, while minimizing the side effects on normal tissue. In our study, the CDH11 inhibitor specifically targeted hybrid EMT tumor cells and activated the cGAS‐STING pathway, Thereby, the induction of apoptosis in these cells simultaneously stimulates immune cell activation. This result is in line with previous findings.^[^ ^50^, ^51^, ^52^ ^]^ Furthermore, the activation of the cGAS‐STING pathway in hybrid EMT tumor cells leads to the release of IFN‐β, which may stimulate immune cell activation and results in the elimination of other tumor cells. We also consider further investigation into the activation of the immune response within the tumor following CDH11 inhibition‐mediated cGAS‐STING activation. Our subsequent research project aimed to reconstitute the immune system of severely immunodeficient mice using human peripheral blood cells, enabling a comprehensive exploration of the impact and underlying mechanisms of CDH11 inhibitors on immune system activation. This study is expected to provide a solid theoretical foundation for future clinical applications of CDH11 inhibitors.

Previous studies on EMT in breast cancer have suggested that this transition is a continuous and dynamic phenomenon involving multiple intermediate cell types. However, owing to the limitations in sample size and sequencing depth, future research should explore additional intermediate cell populations to identify the key molecules that could potentially modulate this intricate process. Moreover, the metabolic dysregulation of BCAA in hybrid EMT cells leads to reduced R‐CoA levels, which are related to TAC. Studies have demonstrated the potential activation of the mTOR pathway in BCAA metabolism, which is involved in tumor development. Further investigations are warranted to elucidate the underlying mechanisms by which BCAA contribute to SACC tumor progression.

The presence of lung metastases in SACC is a pivotal prognostic determinant, and it is necessary to identify drugs capable of inhibiting or retarding its progression. Previous studies have elucidated the tumor‐suppressive effects of CXB via COX2 suppression. Moreover, CDH11 is a signature molecule of hybrid EMT cells in SACC, and our findings reveal that CXB exerts inhibitory effects on SACC lung metastasis by interacting with CDH11. Hybrid EMT cells are present in various tumors, and whether CXB can inhibit lung metastasis in other tumors remains unclear. CXB, a Food and Drug Administration‐approved clinical agent renowned for its favorable safety profile, prompted us to consider its potential for long‐term use as a preventive measure against pulmonary metastasis. However, prolonged administration may impose a cardiac burden, necessitating further investigations to determine the optimal dose.

## 4 Experimental Section

### Ethics Statement

The use of clinical samples was authorized by the Ethics Committee of Peking University School and Hospital of Stomatology (Beijing, China; permit numbers: PKUSSIRB‐201522040 and PKUSSIRB‐202169170). The process of acquiring tumor tissue in this study was approved by the patients with SACC. All animal interventions and protocols were approved by the Institutional Animal Care and Use Committee of Peking University (permit number: PURB‐LA2024066).

### Patients and Sample Collection

SACC tumor specimens were obtained from five patients, along with a non‐infiltrated SMG. These samples were collected from Peking University Stomatological Hospital and confirmed as SACC by the Department of Pathology prior to surgical removal. The Ethics Review Committee of Peking University approved the collection protocol. Subsequently, the acquired tumor tissues were divided into two equal portions. One portion was dissociated using the single‐cell suspension method for scRNA‐seq, and the other portion was embedded in an optimal cutting temperature compound and frozen to facilitate spatial transcriptomic analysis.

### Cell Lines and Transfection

The SACC‐83 cell line, derived from a patient's sublingual gland, was cultured in Roswell Park Memorial Institute (RPMI) 1640 medium (Gibco, 51 170 712, USA) supplemented with 10% fetal bovine serum and incubated at 37 °C in a humidified atmosphere of 95% air and 5% CO_2_. The cell line authentication was conducted using short tandem repeat polymerase chain reaction, and all cultures were negative for mycoplasma contamination. CDH11 overexpression and vector plasmids were obtained from GeneChem (Genechem, China), whereas CDH11 siRNA and control siRNA were purchased from Ribo (Ribo, China). The efficiency of overexpression and knockdown was evaluated using qRT‐PCR and western blot analyses. For transient transfection, plasmids and siRNA prepared according to the manufacturer's instructions were combined with Lipofectamine 8000 (Beyotime, C0533, China) for transfection into cells for 24–48 h, and the transfection efficiency was confirmed by qRT‐PCR and western blotting.

### Tissue Dissociation

Fresh samples were preserved in Tissue Storage Solution (Miltenyi Biotec, 130‐100‐008, USA) on ice and promptly transferred to the laboratory. After rinsing with phosphate buffered saline, enzymatic digestion was performed to obtain single‐cell suspensions. Fresh specimens with attached tissues removed were rinsed with 5 mL of pre‐cooled Dulbecco's modified eagle medium (DMEM) (Gibco, 11 965 092, USA). The tissues were then sliced into < 1 mm^3^ fragments and transferred into a 2 mL centrifuge tube. Subsequently, 1 mL of an enzyme suspension was added, containing 15 µL collagenase II, 15 µL collagenase IV, 10 µL neutral protease/dispase, 5 µL hyaluronidase, 6 µL DNase I, and 1 µL calcium‐magnesium‐zinc buffer. The mix was dissociated at 37 °C for 30 min, with observation every 5 min. The primary single‐cell suspension was filtered through a 70‐µm cell sieve into a 50‐mL centrifuge tube, which was then rinsed with 4 mL of pre‐cooled DMEM. The cell suspension was centrifuged at 500 × g for 5 min at 4 °C, and the supernatant was removed. The cells were re‐suspended with 5 mL pre‐cooled DMEM and centrifuged again at 4 °C at 400 g for 5 min. The liquid was aspirated and 1 mL of pre‐cooled DMEM was added to the precipitate to re‐suspend the cells. The cell state observations were then conducted, and cell concentration, viability, and fragmentation rate were recorded. Trypan blue staining was performed to ensure that the survival rate exceeded 80%. Approximately 10000 cells per sample were collected for single‐cell transcriptome sequencing.

### Single‐Cell Library Preparation and Sequencing

The Single‐Cell 3′ Library Kit v3 (10x Genomics) was employed for single‐cell transcriptome amplification and library preparation following the established protocols.^[^ ^57^, ^58^ ^]^ The sorted single‐cell suspension was loaded onto a microfluidic chip provided by 10x Genomics to generate a complementary DNA (cDNA) library. The library was prepared and sequenced across six lanes using the Illumina NovaSeq 6000 system (Illumina Inc., San Diego, CA, USA).

### Preprocessing of single‐cell RNA sequencing Data

The scRNA‐seq gene expression library was generated using the STAR algorithm with the CellRanger count function (10x Genomics, version 4), as previously described.^[^ ^57^, ^58^ ^]^ The resulting gene expression matrices were processed with version 3.1.4 of the Seurat R package,^[^ ^59^ ^]^ which requires genes to be expressed in at least 10 cells within a sample. Low‐quality cells were eliminated based on specific criteria, including unique multiplex index (UMI) counts, number of expressed genes, and mitochondrial gene percentage. Doublets were identified and removed using the DoubletFinder package in R software. The remaining high‐quality single‐cell transcriptome expression matrices were integrated using the Harmony package in R and normalized using the total cellular UMI count, while scaling them through regression against the total cellular UMI count and percentage of mitochondrial genes (scale factor = 1 × 10^4^). UMAP techniques were used to reduce the dimensions and visualize gene expression. Bioinformatics analysis was conducted using Omicsmart, an interactive online platform designed for real‐time data analysis (http://www.omicsmart.com).

### Determination of Cell Type

DEG in each cell sub‐cluster were determined using the FindAllMarker function, with default parameters set by Seurat, to identify specific genes that exhibited significant expression differences. The cell types and subtypes were annotated based on the expression levels of well‐established canonical marker genes specific to each cell type. The cell sub‐clusters exhibiting similar gene expression patterns were classified as belonging to the same cell type.

### Differentially expressed genes Analysis

The expression levels of each gene in a given cluster were compared with those in the remaining cells using the Wilcoxon rank‐sum test.^[^ ^60^ ^]^ Significantly upregulated genes were identified based on three criteria: a minimum 1.28‐fold increase in expression within the targeted cluster, expression in > 25% of cells belonging to the targeted cluster, and a significance level of *P *< 0.05.

### Pathway Analysis

The DEG with a Q‐value ≤ 0.05 were used for GO enrichment analysis.^[^ ^61^ ^]^The Q‐value represents the adjusted *P*‐value after the false discovery rate correction. To identify distinct enriched GO terms among the sub‐clusters, the cluster functions were compared using the ClusterProfiler R package. The KEGG database was used to perform the pathway significant enrichment analysis,^[^ ^62^ ^]^ and significantly enriched pathways with a Q‐value ≤ 0.05 were identified after multiple testing correction. Additionally, GSVA and GSEA was employed with gene sets from MsigDB to identify enriched pathways and cellular processes in distinct clusters.^[^ ^63^ ^]^

### Cell Cycle Analysis

The cell cycle score for each cell was assigned using the Seurat R package based on the expression levels of marker genes in different phases. This included 100 marker genes for G1/S, 113 for S, 133 for G2/M, 151 for M, and 10 for M/G1.^[^ ^64^ ^]^ The cells with the highest scores (< 0.3) were identified as quiescent cells.^[^ ^14^ ^]^

### Single‐Cell Copy Number Variation Prediction

The prediction of single‐cell CNV involves inferring relative gene expression levels by establishing an expression baseline using normal samples, and subsequently subtracting the gene expression level in each cell from this baseline value. Additionally, a 100‐gene window was employed on the chromosome to predict CNV events within the chromosomal region of a single cell based on relative gene expression. The methods used were those described by Puram et al.,^[^ ^65^ ^]^ and Gene Denovo Biotechnology provided the analysis services.

### Spatial Transcriptome

The tissues were first frozen in isopentane, embedded in Optimal cutting temperature compound, cryosectioned using a cryostat to generate appropriately sized sections for visible spatial slides, and frozen throughout the process. Only samples with an RNA integrity number > 8.0 were used for sequencing. The sections were then placed on the Visium spatial slide within the capture area. Finally, HE staining and bright‐field microscopy were performed. The Visium spatial tissue optimization (TO) slides contained eight mRNA capture areas defined by etched frames, each containing oligonucleotides. Tissue sections measuring 10 µm from the same sample were placed onto these capture areas on the TO slides. The sections were fixed, stained, and permeabilized for 3, 6, 12, 18, 24, or 30 min. The optimal permeabilization time was determined by obtaining the maximum fluorescence signal with minimal diffusion. Therefore, a permeabilization time of 24 min was selected as the final choice. The subsequent steps involved a formal 24 min permeabilization process, followed by cDNA amplification, library construction, and RNA‐seq. Bioinformatics analysis was conducted using Omicsmart, an interactive online platform designed for real‐time data analysis (http://www.omicsmart.com).

### RNA sequencing and Analysis

Total RNA was isolated using TRIzol reagent. The quality of RNA was assessed using Agilent Bioanalyzer 2100 (Agilent Technologies, Santa Clara, CA, USA). Total RNA was purified using the RNAClean XP Kit (Beckman Coulter, Brea, CA, USA) and RNase‐Free DNase Set (QIAGEN, Hilden, Germany). Sequencing libraries were prepared using the Illumina TruSeq RNA sample preparation Kit (Illumina, San Diego, CA, USA). Paired‐end sequencing of the libraries was performed using an Illumina NovaSeq 600 system. Bioinformatics analysis was conducted using Omicsmart, an interactive online platform designed for real‐time data analysis (http://www.omicsmart.com).

### Cell Proliferation, Migration, and Invasion Assays

Cell viability was assessed using CCK‐8 reagent (Dojindo Laboratories, CK40, Kumamoto, Japan) according to the manufacturer's instructions. The cells were seeded at a density of 2000 cells per well in standardized polystyrene plates, with 100 µL culture medium added to each well. After culturing for specific time intervals (ranging from 0 to 96 h), the absorbance values were measured using an ELx808 absorbance microplate reader (BioTek, USA).

For transwell migration and invasion analysis, the cells were seeded into inserts with an 8.0‐µm pore size (Millipore, CLS3464, USA) without Matrigel coating for migration or with Matrigel coating (BD Bioscience, 356 234, USA) for invasion. Subsequently, 8 × 10^4^ cells were plated in serum‐free medium and incubated for 18 h. Non‐migratory and non‐invasive cells on the upper surface of the insert were gently wiped off using a swab, whereas migratory and invasive cells that reached the bottom chamber through the pores were fixed with 95% ethanol and stained with 1% crystal violet. Finally, the cells were quantified using a BX51 microscope (Olympus, Tokyo, Japan).

### Immunoprecipitation

For the immunoprecipitation experiments, the cells were lysed using immunoprecipitation lysis buffer (Beyotime, P0037, China) and centrifuged at 4 °C for 10 min. The resulting supernatant was incubated overnight at 4 °C with the designated primary antibody(5 µg). After appropriate washing steps, Protein A+G agarose gel was added to the solution and incubated for 4 h at room temperature. Unbound proteins were removed by thoroughly rinsing the immune complexes, whereas bound immune complexes were eluted from the agarose gel using a sodium dodecyl sulfate–polyacrylamide gel electrophoresis (SDS‐PAGE) loading buffer for subsequent detection. The specific experimental procedures were performed in accordance with the manufacturer's instructions. Western blot analysis was performed to confirm the presence of target proteins within this experiment. A successful sample was subjected to the excision of bands from the gel for identification, using mass spectrometry, and the intersection of the two outcomes was considered.

### Branched‐Chain Amino Acid (BCAA) Assay

SACC‐83 cells were seeded into six‐well plates and transfected with DNA plasmids or siRNA 24 h later. After a 48 h incubation period, the cells were lysed, and the levels of BCAA were quantified following the manufacturer's instructions (Sigma‐Aldrich, MAK003, USA). To dilute, approximately 10 µL of cell lysate was added to each well of a 96‐well plate, and the final volume was adjusted to 50 µL using an assay buffer. Subsequently, 46 µL of assay buffer, along with 2 µL of BCAA enzyme mix and substrate mix, was added to each well to reach a final volume of 100 µL per well. The blanks were prepared by mixing the cell lysate with the assay buffer and substrate mix, while omitting the enzyme. The absorbance values of the blank samples were subtracted from those of cell lysates. A standard curve was generated using pure leucine as a reference. The readings were acquired using an enzyme immunoassay analyzer.

### Western Blot Analysis

Western blot analysis was performed according to a standardized protocol. Briefly, 40 µg of protein derived from cells or exosomes was resolved by SDS‐PAGE and transferred onto polyvinylidene difluoride membranes. Following membrane blocking, the primary antibodies listed below were employed: anti‐BCKDHA (Proteintech, 30028; 1:2000, USA), anti‐BCKDHB (Proteintech, 13685; 1:2000, USA), anti‐DBT (Singon, D163672, 1:1000, China), anti‐phosphor‐BCKDH‐E1α (Cell Signaling Technology, 40368S, 1:1000, USA), anti‐phospho‐stimulator of interferon response cGAMP interactor (p‐STING) (Cell Signaling Technology, 19781, 1:1000, USA), anti‐STING (Cell Signaling Technology, 13647, 1:1000, USA), anti‐phospho‐TANK binding kinase 1 (p‐TBK1) (Cell Signaling Technology, 5483, 1:1000, USA), anti‐TBK1 (Cell Signaling Technology, 3504, 1:1000, USA), anti‐phospho‐IRF3 (Cell Signaling Technology, 37829, 1:1000, USA), anti‐IRF3 (Cell Signaling Technology, 4302, 1:1000, USA), anti‐GAPDH (ZSGB‐BIO, TA‐08, 1:1000, China), and anti‐CDH11 (ThermoFisher Scientific, 32–1700, 1:1000, USA).

### Quantitative reverse transcription polymerase chain reaction

Total RNA was isolated using the Trizol reagent. The quality of RNA was assessed using an Agilent Bioanalyzer 2100 (Agilent Technologies, Santa Clara, CA, USA). The qRT‐PCR primers used in this study are listed in Table S2 (Supporting Information). The quantification of mRNA expression was performed using the FastStart Universal SYBR Green Master (ROX) reagent (Roche, 4 913 850 001, USA) on an ABI 7500 Sequence Detection System for qRT‐PCR analysis. The mRNA expression levels of the genes of interest were normalized according to GAPDH, and the results were presented as a fold change using the ΔΔCt method with the control set as one. Survival was analyzed using the Kaplan‐Meier method, and differences were evaluated using the log‐rank test.

### Flow Cytometric Sorting and Analysis

The SACC‐83 cells were incubated with antibodies recognizing ITGA2 (Biolegend, 359 309, 5 µL per test, USA) and PDGFRα (Biolegend, 323 505, 5 µL per test, USA) for 30 min at 4 °C. Subsequently, cells were separated and collected for culture, including ITGA2^+^PDGFRα^+^, ITGA2^+^PDGFRα^−^, ITGA2^−^PDGFRα^+^, and ITGA2^−^PDGFRα^−^. Subsequently, the cell suspension was cultured in a low‐adhesion culture dish. After 48 h, the cells were harvested and analyzed using an FITC‐Annexin‐V/PI apoptosis detection kit (Beyotime, C1062S, China), following the manufacturer's instructions, for flow cytometric quantification of apoptotic cells.

### Cellular Thermal Shift Assay

The ability of the compounds to interact with and stabilize the target in intact cells was assessed according to the protocol described by Molina et al.^[^ ^66^ ^]^ Briefly, 10 million cells were treated with media containing DMSO, celecoxib (CXB), dimethyl celecoxib (DMC), or SD‐133 (at IC_50_ doses) for 6 h. After treatment, the cells were detached using trypsin, collected by centrifugation, and re‐suspended in Phosphate Buffer Saline(PBS). The cell suspension was divided into five PCR tubes and subjected to heating at temperatures ranging from 53 °C to 66 °C for a period of 3 min each. Subsequently, the cells were lysed using liquid nitrogen, followed by two freeze‐thaw cycles. The precipitated proteins were separated from the soluble fraction by centrifugation at 17, 000 × g for 20 min. The soluble proteins present in the supernatant were stored at ‐80 °C until western blot analysis could be performed. Equal amounts of protein samples were loaded onto SDS‐PAGE gels, which were then transferred onto nitrocellulose membranes for subsequent analysis using the CDH11 antibody at a concentration ratio of 1:1000. The protein expression levels on western blots were quantified by densitometry using ImageJ software.

### Surface Plasmon Resonance

The Biacore 1K Cytiva instrument and CM7 sensor chip were utilized. First, the Recombinant Human His‐Tagged Cadherin‐11 Protein (10065‐H08H, Sino Biological, China) was immobilized. The CM7 chip was activated using the Amine Coupling Kit (35 063, Cytiva, Sweden) per the instructions at a flow rate of 10 µL min^−1^ for 420 sec. Subsequently, the protein was injected at a flow rate of 10 µL min^−1^ with a concentration of 40 µg mL^−1^ for a fixed duration of 600 s + 600 s. Then, the sample was blocked with ethanolamine (35 063, Cytiva) at a rate of 10 µL min^−1^ for 420 s. Finally, CXB, DMC, or SD‐133 were injected at a flow rate of 30 µL min^−1^ with 60 sec for contact and 120 sec for dissociation. Additionally, the temperature was maintained at 25 °C with a concentration ranging from 100 to 3.125 µm, employing a two‐fold gradient reduction method to adjust the concentration of the sample injected. Each injection was repeated 3 times.

### Molecular Docking

Molecular docking was performed using the AutoDock Vina software with a semi‐flexible docking method applied between CDH11 and SD‐133/DMC/CXB. First, the system coordinate file was prepared using AutoDockTools, and the binding area's center and box size were determined. Subsequently, ligand pairs were attached to the receptor active site using AutoDock Vina, and the results were further analyzed.

### ROS Assay

An ROS Assay Kit (Beyotime, S0033S, China) or Dihydroethidium (DHE) (Beyotime, S0063, China) was used to conduct the experiment according to the manufacturer's instructions, and subsequent cell stimulation was performed under specific conditions. DCFH‐DA was diluted in a serum‐free medium at a ratio of 1:1000 to achieve a final concentration of 10 mmol L^−1^. Subsequently, the cells were suspended in the diluted DCFH‐DA solution at a concentration ranging from 20 million mL^−1^ and incubated in a cell incubator set at 37 °C for 20 min. The suspension was inverted and mixed every 3–5 min to ensure a complete contact between the probe and the cells. To eliminate any residual DCFH‐DA that did not enter the cells, three rinses with serum‐free cell culture solution were performed. The detection process of DHE is the same as that of DCFH‐DA, but the cells were typically incubated with 3 µmol of Dihydroethidium at 37 °C for a duration of 30 min. Finally, flow cytometry was performed to detect ROS production after collecting the treated cells.

### Cytosolic Double‐Stranded DNA Staining

After treatment, the SACC‐83 cells were incubated in culture media supplemented with a 200‐fold diluted solution of PicoGreen (P11496, 1:200, USA), a double‐stranded DNA stain. Additionally, MitoTracker (ThermoFisher Scientific, M7512,500n M, USA), another mitochondrial DNA staining agent, was added to the culture medium. Following the 1 h incubation period, the cells were fixed with 4% paraformaldehyde for 10 min. Subsequently, the cells were rinsed thrice with PBS and stained with DAPI Staining Solution (ZSGB‐BIO, 9556, China). Finally, the staining was visualized and evaluated using Leica SP5X laser‐scanning confocal microscope (Leica, Wetzlar, Germany).

### Comet Assays

Single‐cell gel electrophoresis comet assays were performed using the SCGE assay kit (Beyotime, C2041S, China). Following treatment, 1 × 10^6^ mL^−1^ cells were mixed with low melting point agarose at a volume ratio of 1:8. The slides were first incubated in a pre‐chilled lysis solution for 2 h, and then in a pre‐chilled alkaline solution for 30 min. Electrophoresis was performed at 25 V in a Tris‐Borate‐EDTA buffer for 20 min. The comets were stained with Propidium Iodide for 30 min and imaged.

### BCAA Conditioned Medium

The BCAA‐deprived RPMI 1640 medium obtained from Yuchun Biology lacks L‐leucine, L‐isoleucine, and L‐valine. To create the BCAA‐added 1640 medium, 50 mg L^−1^ L‐leucine (Aladdin, 61‐90‐5, China), 50 mg L^−1^ L‐isoleucine (Aladdin, 73‐32‐5, China), and 20 mg L^−1^ L‐valine (Aladdin, 72‐18‐4, China) were added. The SACC‐83 cells were cultured in both BCAA‐deprived and BCAA‐added 1640 media for a culture period of 24 h, and the subsequent experiments were conducted.

### In Vivo Experiment

Female NOD‐SCID mice (6–8 weeks old) were obtained from Vital River Laboratories (Beijing, China). The animal experiments were approved by the Peking University Institutional Animal Care and Use Committee (permit number: PURB‐LA2024066) and were performed in accordance with the guidelines for animal experiments. To assess the therapeutic efficacy of the three drugs on the pulmonary metastasis of SACC in vivo, 2 × 106 SACC‐83 cells were injected into the tail veins of NOD‐SCID mice (*n* = 6 per group). The drug was dissolved in a solution containing 0.5% methylcellulose and 0.025% Tween 80. The medication was initiated the following day. CXB/DMC/SD‐133 was administered intraperitoneally at 5 mg kg^−1^,^[^ ^36^, ^67^ ^]^ once every 2 days for 8 weeks. After 8 weeks, the mice were sacrificed and their lungs were collected for quantification and hematoxylin and eosin staining. To assess the cytotoxicity of the three drugs, HE staining was performed on the major organs of mice following treatment, including the heart, liver, spleen, and kidneys. The tissue sections were then photographed using a BX51 microscope (Olympus, Tokyo, Japan).

### Immunohistochemical Staining

Immunohistochemical staining was performed on 5 µm‐thick sections obtained from samples fixed in 4% paraformaldehyde and embedded in paraffin. The sections were incubated overnight at 4 °C with anti‐IFN‐beta (27 506, 1:100, Proteintech, USA) as the primary antibody. Subsequently, horseradish peroxidase‐labeled secondary antibody (PV‐6001, ZSGB‐BIO, China) and hematoxylin were utilized for sample staining. Immuno‐staining and analysis were conducted on at least three sections per lesion. All images were captured using an Olympus BX51 microscope (Olympus).

### Human Peripheral Blood Mononuclear Cell Separation

Peripheral blood samples (20 mL) were donated by anonymous healthy volunteers and collected in heparin‐containing vessels. The samples were then diluted 1:1 with phosphate buffer saline (PBS). The diluted blood samples were carefully dripped along the wall of a new 50 mL sterile centrifuge tube containing Human Peripheral Blood Lymphocyte Separation Medium (C0025, Beyotime, China) at a 45‐degree angle. The mixture was centrifuged at 650 ×*g* for 30 min using a horizontal rotor centrifuge with the acceleration set to 3, brakes turned off, and temperature set to 20 °C. After removing as much upper plasma as possible, the peripheral blood mononuclear cell layer was transferred into another centrifuge tube. To resuspend the cells, 10 mL PBS was added followed by centrifugation at 250 ×*g* for 10 min. The supernatant was discarded and 5 mL of PBS was added before starting two more rounds of centrifugation under same conditions until isolated mononuclear cells were obtained. Finally, 0.5 mL PBS was added for subsequent experiments.

### Co‐Culturing SACC‐83 Cells with Mononuclear Cells

Briefly, 1 × 10^6^ SACC‐83 cells were seeded in a dish with a diameter of 6 cm and cultured for 12 h until fully adhered. Then, the cells were treated with media containing DMSO, CXB, DMC, or SD‐133 (at IC_50_ doses) for 12 h. Subsequently, sorted mononuclear cells (1 × 107 cells per dish), including lymphocytes and monocytes, were added in a final volume of 4 mL. The co‐cultures were incubated with 5% CO_2_ at 37 °C for 24 h. Similar numbers of immune cells cultured alone served as references. The supernatant from the culture dish was collected and subsequently centrifuged at 450 ×*g* for 5 min to isolate lymphocytes for flow cytometric analysis.

### Flow Cytometry for Cytotoxic T Lymphocytes

Single‐cell suspensions were stained with Fixable Viability Dyes (65‐0868, Thermo Fisher Scientific, USA), as instructed, and were incubated with anti‐CD8 antibodies (5 uL per test; 344 705, BioLegend, USA) for 30 min, according to the manufacturer instructions. Subsequently, the cell membrane was permeabilized using the Intracellular Fixation & Permeabilization Buffer Set (88‐8824, Thermo Fisher Scientific). Anti‐perforin and anti‐granzyme B antibodies (5 ul per test; 308 103 and 372 203, respectively; BioLegend) were added and incubated at room temperature for 30 min in a light‐free environment. After adding 2 mL of the Intracellular Fixation & Permeabilization Buffer, centrifugation was performed twice at room temperature at 450 × *g* for 5 min each. The supernatant was then discarded. The cells were resuspended in an appropriate volume of Flow Cytometry Staining Buffer (00‐4222, Thermo Fisher Scientific). Finally, the sample was loaded into a flow cytometer for analysis.

### Statistical Analyses

The data were analyzed using Prism software (GraphPad Software 8, USA). Numerical data were presented as mean ± SEM from at least three independent experiments, unless otherwise specified. Statistical significance was determined using appropriate tests, including a two‐tailed t‐test or one‐way analysis of variance. A *P*‐value < 0.05 indicated statistical significance.

### Ethics Approval Statement

The use of clinical samples was authorized by the Ethics Committee of Peking University School and Hospital of Stomatology (Beijing, China; permit numbers: PKUSSIRB‐201522040 and PKUSSIRB‐202169171). All animal interventions and protocols were approved by the Institutional Animal Care and Use Committee of Peking University (permit number: PURB‐LA2024066).

### Patient Consent Statement

The process of acquiring tumor tissue in this study was approved by the patients.

## Conflict of Interest

The authors declare no conflict of interest.

## Supporting information



Supporting Information

Supplemental Table 1

## Data Availability

RNA‐seq data analyzed in this study can be obtained from NCBI (https://submit.ncbi.nlm.nih.gov/subs/sra/), PRJNA1131155 and PRJNA1131177. scRNA‐seq data analyzed in this study can be obtained from NCBI (https://submit.ncbi.nlm.nih.gov/subs/sra/), PRJNA1131759. ST sequencing data analyzed in this study can be obtained from NCBI (https://submit.ncbi.nlm.nih.gov/subs/sra/), PRJNA1133240.

## References

[1] A. S. Ho , A. Ochoa , G. Jayakumaran , A. Zehir , C. Valero Mayor , J. Tepe , V. Makarov , M. G. Dalin , J. He , M. Bailey , M. Montesion , J. S. Ross , V. A. Miller , L. Chan , I. Ganly , S. Dogan , N. Katabi , P. Tsipouras , P. Ha , N. Agrawal , D. B. Solit , P. A. Futreal , A. K. El Naggar , J. S. Reis‐Filho , B. Weigelt , A. L. Ho , N. Schultz , T. A. Chan , L. G. Morris , J. Clin. Invest. 2019, 129, 4276. 31483290 10.1172/JCI128227 PMC6763222

[2] K. Bjørndal , A. Krogdahl , M. H. Therkildsen , J. Overgaard , J. Johansen , C. A. Kristensen , P. Homøe , C. H. Sørensen , E. Andersen , T. Bundgaard , H. Primdahl , K. Lambertsen , L. J. Andersen , C. Godballe , Oral Oncol. 2011, 47, 677. 21612974 10.1016/j.oraloncology.2011.04.020

[3] V. L. Vander Poorten , A. J. Balm , F. J. Hilgers , I. B. Tan , B. M. Loftus‐Coll , R. B. Keus , A. A. Hart , Cancer 1999, 85, 2255. 10326706 10.1002/(sici)1097-0142(19990515)85:10<2255::aid-cncr22>3.3.co;2-4

[4] L. Ciccolallo , L. Licitra , G. Cantú , G. Gatta , Oral Oncol. 2009, 45, 669. 19095489 10.1016/j.oraloncology.2008.10.010

[5] B. Xu , E. Drill , A. Ho , A. Ho , L. Dunn , C. N. Prieto‐Granada , T. Chan , I. Ganly , R. Ghossein , N. Katabi , Am. J. Surg. Pathol. 2017, 41, 1422. 28719465 10.1097/PAS.0000000000000918 PMC5597477

[6] R. Ferrarotto , Y. Mitani , D. J. McGrail , K. Li , T. V. Karpinets , D. Bell , S. J. Frank , X. Song , M. E. Kupferman , B. Liu , J. J. Lee , B. S. Glisson , J. Zhang , J. C. Aster , S.‐Y. Lin , P. A. Futreal , J. V. Heymach , A. K. El‐Naggar , Clin. Cancer Res. 2020, 27, 852. 33172898 10.1158/1078-0432.CCR-20-1192 PMC7854509

[7] B. Bakir , A. M. Chiarella , J. R. Pitarresi , A. K. Rustgi , Trends Cell Biol. 2020, 30, 764. 32800658 10.1016/j.tcb.2020.07.003 PMC7647095

[8] D. Bartis , N. Mise , R. Y. Mahida , O. Eickelberg , D. R. Thickett , Thorax 2014, 69, 760. 24334519 10.1136/thoraxjnl-2013-204608

[9] M. A. Nieto , R. Y. Huang , R. A. Jackson , J. P. Thiery , Cell 2016, 166, 21. 27368099 10.1016/j.cell.2016.06.028

[10] I. Pastushenko , A. Brisebarre , A. Sifrim , M. Fioramonti , T. Revenco , S. Boumahdi , A. Van Keymeulen , D. Brown , V. Moers , S. Lemaire , S. De Clercq , E. Minguijón , C. Balsat , Y. Sokolow , C. Dubois , F. De Cock , S. Scozzaro , F. Sopena , A. Lanas , N. D'Haene , I. Salmon , J. C. Marine , T. Voet , P. A. Sotiropoulou , C. Blanpain , Nature 2018, 556, 463. 29670281 10.1038/s41586-018-0040-3

[11] I. Pastushenko , C. Blanpain , Trends Cell Biol. 2019, 29, 212. 30594349 10.1016/j.tcb.2018.12.001

[12] A. L. Ji , A. J. Rubin , K. Thrane , S. Jiang , D. L. Reynolds , R. M. Meyers , M. G. Guo , B. M. George , A. Mollbrink , J. Bergenstrahle , L. Larsson , Y. Bai , B. Zhu , A. Bhaduri , J. M. Meyers , X. Rovira‐Clave , S. T. Hollmig , S. Z. Aasi , G. P. Nolan , J. Lundeberg , P. A. Khavari , Cell 2020, 182, 497. 32579974 10.1016/j.cell.2020.05.039 PMC7391009

[13] Z. Chen , L. Zhou , L. Liu , Y. Hou , M. Xiong , Y. Yang , J. Hu , K. Chen , Nat. Commun. 2020, 11, 5077. 33033240 10.1038/s41467-020-18916-5 PMC7545162

[14] C. Neftel , J. Laffy , M. G. Filbin , T. Hara , M. E. Shore , G. J. Rahme , A. R. Richman , D. Silverbush , M. L. Shaw , C. M. Hebert , J. Dewitt , S. Gritsch , E. M. Perez , L. N. Gonzalez Castro , X. Lan , N. Druck , C. Rodman , D. Dionne , A. Kaplan , M. S. Bertalan , J. Small , K. Pelton , S. Becker , D. Bonal , Q. D. Nguyen , R. L. Servis , J. M. Fung , R. Mylvaganam , L. Mayr , J. Gojo , et al., Cell 2019, 178, 835. 31327527 10.1016/j.cell.2019.06.024 PMC6703186

[15] Y. Wu , S. Yang , J. Ma , Z. Chen , G. Song , D. Rao , Y. Cheng , S. Huang , Y. Liu , S. Jiang , J. Liu , X. Huang , X. Wang , S. Qiu , J. Xu , R. Xi , F. Bai , J. Zhou , J. Fan , X. Zhang , Q. Gao , Cancer Discovery 2021, 12, 134. 34417225 10.1158/2159-8290.CD-21-0316

[16] D. Bell , D. Roberts , M. Kies , P. Rao , R. S. Weber , A. K. El‐Naggar , Cancer 2010, 116, 5749. 20824717 10.1002/cncr.25541 PMC2998592

[17] R. Ferrarotto , J. V. Heymach , B. S. Glisson , Curr. Opin. Oncol. 2016, 28, 195. 26974847 10.1097/CCO.0000000000000280

[18] A. Butler , P. Hoffman , P. Smibert , E. Papalexi , R. Satija , Nat. Biotechnol. 2018, 36, 411. 29608179 10.1038/nbt.4096 PMC6700744

[19] I. Korsunsky , N. Millard , J. Fan , K. Slowikowski , F. Zhang , K. Wei , Y. Baglaenko , M. Brenner , P. R. Loh , S. Raychaudhuri , Nat. Methods 2019, 16, 1289. 31740819 10.1038/s41592-019-0619-0 PMC6884693

[20] D. Aran , A. P. Looney , L. Liu , E. Wu , V. Fong , A. Hsu , S. Chak , R. P. Naikawadi , P. J. Wolters , A. R. Abate , A. J. Butte , M. Bhattacharya , Nat. Immunol. 2019, 20, 163. 30643263 10.1038/s41590-018-0276-y PMC6340744

[21] X. Zhang , Y. Lan , J. Xu , F. Quan , E. Zhao , C. Deng , T. Luo , L. Xu , G. Liao , M. Yan , Y. Ping , F. Li , A. Shi , J. Bai , T. Zhao , X. Li , Y. Xiao , Nucleic Acids Res. 2019, 47, D721. 30289549 10.1093/nar/gky900 PMC6323899

[22] C. Hu , T. Li , Y. Xu , X. Zhang , F. Li , J. Bai , J. Chen , W. Jiang , K. Yang , Q. Ou , X. Li , P. Wang , Y. Zhang , Nucleic Acids Res. 2023, 51, D870. 36300619 10.1093/nar/gkac947 PMC9825416

[23] C. Clarke , J. Sandle , S. R. Lakhani , J. Mammary Gland Biol. Neoplasia 2005, 10, 273. 16807806 10.1007/s10911-005-9587-3

[24] L. H. Xu , F. Zhao , W. W. Yang , C. W. Chen , Z. H. Du , M. Fu , X. Y. Ge , S. L. Li , Int. J. Oncol. 2019, 54, 1579. 30896785 10.3892/ijo.2019.4754 PMC6438425

[25] J. Massé , C. Truntzer , R. Boidot , E. Khalifa , G. Pérot , V. Velasco , L. Mayeur , C. Billerey‐Larmonier , L. Blanchard , H. Charitansky , I. Soubeyran , R. Iggo , L. Arnould , G. MacGrogan , Mod. Pathol. 2020, 33, 1041. 31857685 10.1038/s41379-019-0425-3

[26] R. Ferrarotto , Y. Mitani , L. Diao , I. Guijarro , J. Wang , P. Zweidler‐McKay , D. Bell , W. N. William , B. S. Glisson , M. J. Wick , A. M. Kapoun , A. Patnaik , G. Eckhardt , P. Munster , L. Faoro , J. Dupont , J. J. Lee , A. Futreal , A. K. El‐Naggar , J. V. Heymach , J. Clin. Oncol. 2016, 35, 352. 27870570 10.1200/JCO.2016.67.5264 PMC5456373

[27] S. Lamouille , J. Xu , R. Derynck , Nat. Rev. Mol. Cell Biol. 2014, 15, 178. 24556840 10.1038/nrm3758 PMC4240281

[28] F. Du , C.‐X. Zhou , Y. Gao , Ann. Diagn. Pathol. 2016, 22, 12. 27180054 10.1016/j.anndiagpath.2016.03.001

[29] F. Deng , D. Bell , 2022.

[30] R. Rimal , P. Desai , R. Daware , A. Hosseinnejad , J. Prakash , T. Lammers , S. Singh , Adv. Drug Delivery Rev. 2022, 189, 114504. 10.1016/j.addr.2022.114504 35998825

[31] M. Neinast , D. Murashige , Z. Arany , Annu. Rev. Physiol. 2019, 81, 139. 30485760 10.1146/annurev-physiol-020518-114455 PMC6536377

[32] S. Sivanand , M. G. Vander Heiden , Cancer Cell 2020, 37, 147. 32049045 10.1016/j.ccell.2019.12.011 PMC7082774

[33] C. Nie , T. He , W. Zhang , G. Zhang , X. Ma , Int. J. Mol. Sci. 2018, 19, 954. 29570613 10.3390/ijms19040954 PMC5979320

[34] M. Funakoshi‐Tago , T. Shimizu , K. Tago , M. Nakamura , H. Itoh , Y. Sonoda , T. Kasahara , Biochem. Pharmacol. 2008, 76, 662. 18644347 10.1016/j.bcp.2008.06.015

[35] G. R. Sareddy , K. Geeviman , C. Ramulu , P. P. Babu , J. Neurooncol. 2012, 106, 99. 21847707 10.1007/s11060-011-0662-x

[36] S. Assefnia , S. Dakshanamurthy , J. M. Guidry Auvil , C. Hampel , P. Z. Anastasiadis , B. Kallakury , A. Uren , D. W. Foley , M. L. Brown , L. Shapiro , M. Brenner , D. Haigh , S. W. Byers , Oncotarget 2014, 5, 1458. 24681547 10.18632/oncotarget.1538 PMC4039224

[37] C. Sobolewski , N. Legrand , Biomolecules 2021, 11, 1049. 34356673 10.3390/biom11071049 PMC8302000

[38] A. Ablasser , Z. J. Chen , Science 2019, 363, aat8657. 10.1126/science.aat8657 30846571

[39] M. R. Papazian , M. Chow , J. Oliver , A. J. Gordon , A. Jacobson , A. Vaezi , M. Tam , B. Givi , Otolaryngol. Head Neck Surg. 2023, 168, 1411. 36892056 10.1002/ohn.203

[40] L. Feeney , B. Hapuarachi , H. Adderley , S. Rack , D. Morgan , R. Walker , R. Rauch , E. Herz , J. Kaye , K. Harrington , R. Metcalf , Oral Oncol. 2022, 133, 106028. 35952580 10.1016/j.oraloncology.2022.106028

[41] M. Persson , Y. Andrén , J. Mark , H. M. Horlings , F. Persson , G. Stenman , Proc. Natl. Acad. Sci. USA 2009, 106, 18740. 19841262 10.1073/pnas.0909114106 PMC2773970

[42] G. J. Hanna , A. Oneill , J. M. Cutler , M. Flynn , T. Vijaykumar , J. R. Clark , L. J. Wirth , J. H. Lorch , J. C. Park , J. K. Mito , J. G. Lohr , J. Kaufman , N. S. Burr , L. I. Zon , R. I. Haddad , Oral Oncol. 2021, 119, 105366. 34091189 10.1016/j.oraloncology.2021.105366

[43] M. L. Suvà , I. Tirosh , Mol. Cell 2019, 75, 7. 31299208 10.1016/j.molcel.2019.05.003

[44] Y. Lei , R. Tang , J. Xu , W. Wang , B. Zhang , J. Liu , X. Yu , S. Shi , J. Hematol. Oncol. 2021, 14, 91. 34108022 10.1186/s13045-021-01105-2 PMC8190846

[45] X. Liu , J. Li , B. L. Cadilha , A. Markota , C. Voigt , Z. Huang , P. P. Lin , D. D. Wang , J. Dai , G. Kranz , A. Krandick , D. Libl , H. Zitzelsberger , I. Zagorski , H. Braselmann , M. Pan , S. Zhu , Y. Huang , S. Niedermeyer , C. A. Reichel , B. Uhl , D. Briukhovetska , J. Suárez , S. Kobold , O. Gires , H. Wang , Sci. Adv. 2019, 5, aav4275. 10.1126/sciadv.aav4275 PMC6584608 31223646

[46] L. Simonneau , M. Kitagawa , S. Suzuki , J. P. Thiery , Cell Adhes. Commun. 1995, 3, 115. 7583005 10.3109/15419069509081281

[47] I. Peran , S. Dakshanamurthy , M. D. McCoy , A. Mavropoulos , B. Allo , A. Sebastian , N. R. Hum , S. C. Sprague , K. A. Martin , M. J. Pishvaian , E. E. Vietsch , A. Wellstein , M. B. Atkins , L. M. Weiner , A. A. Quong , G. G. Loots , S. S. Yoo , S. Assefnia , S. W. Byers , Gastroenterology 2021, 160, 1359. 33307028 10.1053/j.gastro.2020.11.044 PMC7956114

[48] A. Sebastian , N. R. Hum , K. A. Martin , S. F. Gilmore , I. Peran , S. W. Byers , E. K. Wheeler , M. A. Coleman , G. G. Loots , Cancers (Basel) 2020, 12, 1307. 32455670 10.3390/cancers12051307 PMC7281266

[49] B. T. Zheng , Q. L. Li , T. Lan , J. Xie , Y. G. Lu , D. L. Zheng , B. H. Su , Onco Targets Ther. 2021, 14, 4211. 34295163 10.2147/OTT.S298614 PMC8291966

[50] R. Falahat , A. Berglund , R. M. Putney , P. Perez‐Villarroel , S. Aoyama , S. Pilon‐Thomas , G. N. Barber , J. J. Mulé , Proc. Natl. Acad. Sci. USA 2021, 118, 2013598118. 10.1073/pnas.2013598118 PMC8053941 33827917

[51] J. Hu , F. J. Sánchez‐Rivera , Z. Wang , G. N. Johnson , Y. J. Ho , K. Ganesh , S. Umeda , S. Gan , A. M. Mujal , R. B. Delconte , J. P. Hampton , H. Zhao , S. Kottapalli , E. de Stanchina , C. A. Iacobuzio‐Donahue , D. Pe'er , S. W. Lowe , J. C. Sun , J. Massagué , Nature 2023, 616, 806. 36991128 10.1038/s41586-023-05880-5 PMC10569211

[52] S. Liu , W. Guan , Mediators Inflamm. 2018, 2018, 1 202 797. 10.1155/2018/1202797 PMC6286756 30595664

[53] A. Amouzegar , M. Chelvanambi , J. N. Filderman , W. J. Storkus , J. J. Luke , Cancers (Basel) 2021, 13, 2695. 34070756 10.3390/cancers13112695 PMC8198217

[54] X. Sun , Y. Zhang , J. Li , K. S. Park , K. Han , X. Zhou , Y. Xu , J. Nam , J. Xu , X. Shi , L. Wei , Y. L. Lei , J. J. Moon , Nat. Nanotechnol. 2021, 16, 1260. 34594005 10.1038/s41565-021-00962-9 PMC8595610

[55] F. Meric‐Bernstam , R. F. Sweis , S. Kasper , O. Hamid , S. Bhatia , R. Dummer , A. Stradella , G. V. Long , A. Spreafico , T. Shimizu , N. Steeghs , J. J. Luke , S. M. McWhirter , T. Müller , N. Nair , N. Lewis , X. Chen , A. Bean , L. Kattenhorn , M. Pelletier , S. Sandhu , Clin. Cancer Res. 2023, 29, 110. 36282874 10.1158/1078-0432.CCR-22-2235 PMC11188043

[56] F. Meric‐Bernstam , R. F. Sweis , F. S. Hodi , W. A. Messersmith , R. H. I. Andtbacka , M. Ingham , N. Lewis , X. Chen , M. Pelletier , X. Chen , J. Wu , S. M. McWhirter , T. Müller , N. Nair , J. J. Luke , Clin. Cancer Res. 2022, 28, 677. 34716197 10.1158/1078-0432.CCR-21-1963

[57] L. Ma , M. O. Hernandez , Y. Zhao , M. Mehta , B. Tran , M. Kelly , Z. Rae , J. M. Hernandez , J. L. Davis , S. P. Martin , D. E. Kleiner , S. M. Hewitt , K. Ylaya , B. J. Wood , T. F. Greten , X. W. Wang , Cancer Cell 2019, 36, 418. 31588021 10.1016/j.ccell.2019.08.007 PMC6801104

[58] M. Zhang , L. Wu , Y. Deng , F. Peng , T. Wang , Y. Zhao , P. Chen , J. Liu , G. Cai , L. Wang , J. Wu , X. Chen , Front. Immunol. 2022, 13, 857 025. 10.3389/fimmu.2022.857025 PMC9114878 35603220

[59] T. Stuart , A. Butler , P. Hoffman , C. Hafemeister , E. Papalexi , W. M. Mauck 3rd , Y. Hao , M. Stoeckius , P. Smibert , R. Satija , Cell 2019, 177, 1888. 31178118 10.1016/j.cell.2019.05.031 PMC6687398

[60] J. G. Camp , K. Sekine , T. Gerber , H. Loeffler‐Wirth , H. Binder , M. Gac , S. Kanton , J. Kageyama , G. Damm , D. Seehofer , L. Belicova , M. Bickle , R. Barsacchi , R. Okuda , E. Yoshizawa , M. Kimura , H. Ayabe , H. Taniguchi , T. Takebe , B. Treutlein , Nature 2017, 546, 533. 28614297 10.1038/nature22796

[61] M. Ashburner , C. A. Ball , J. A. Blake , D. Botstein , H. Butler , J. M. Cherry , A. P. Davis , K. Dolinski , S. S. Dwight , J. T. Eppig , M. A. Harris , D. P. Hill , L. Issel‐Tarver , A. Kasarskis , S. Lewis , J. C. Matese , J. E. Richardson , M. Ringwald , G. M. Rubin , G. Sherlock , Nat. Genet. 2000, 25, 25. 10802651 10.1038/75556 PMC3037419

[62] M. Kanehisa , S. Goto , Nucleic Acids Res. 2000, 28, 27. 10592173 10.1093/nar/28.1.27 PMC102409

[63] A. Liberzon , C. Birger , H. Thorvaldsdóttir , M. Ghandi , J. P. Mesirov , P. Tamayo , Cell Syst. 2015, 1, 417. 26771021 10.1016/j.cels.2015.12.004 PMC4707969

[64] E. Z. Macosko , A. Basu , R. Satija , J. Nemesh , K. Shekhar , M. Goldman , I. Tirosh , A. R. Bialas , N. Kamitaki , E. M. Martersteck , J. J. Trombetta , D. A. Weitz , J. R. Sanes , A. K. Shalek , A. Regev , S. A. McCarroll , Cell 2015, 161, 1202. 26000488 10.1016/j.cell.2015.05.002 PMC4481139

[65] S. V. Puram , I. Tirosh , A. S. Parikh , A. P. Patel , K. Yizhak , S. Gillespie , C. Rodman , C. L. Luo , E. A. Mroz , K. S. Emerick , D. G. Deschler , M. A. Varvares , R. Mylvaganam , O. Rozenblatt‐Rosen , J. W. Rocco , W. C. Faquin , D. T. Lin , A. Regev , B. E. Bernstein , Cell 2017, 171, 1611. 29198524 10.1016/j.cell.2017.10.044 PMC5878932

[66] Q. Lu , Y. Zhang , J. Hellner , C. Giannini , X. Xu , J. Pauwels , Q. Ma , W. Dejonghe , H. Han , B. Van de Cotte , F. Impens , K. Gevaert , I. De Smet , J. Friml , D. M. Molina , E. Russinova , Proc. Natl. Acad. Sci. USA 2022, 119, 2118220119. 10.1073/pnas.2118220119 PMC8931322 35254915

[67] A. Suri , X. Sheng , K. M. Schuler , Y. Zhong , X. Han , H. M. Jones , P. A. Gehrig , C. Zhou , V. L. Bae‐Jump , Oncotarget 2016, 7, 39582. 27074576 10.18632/oncotarget.8659 PMC5129955

