# Correction to “Inhibition of CDH11 Activates cGAS‐STING by Stimulating Branched Chain Amino Acid Catabolism and Mitigates Lung Metastasis of Adenoid Cystic Carcinoma”

**DOI:** 10.1002/advs.202504992

**Published:** 2025-05-29

Li RF, Liu S, Gao Q, et al. Inhibition of CDH11 Activates cGAS‐STING by Stimulating Branched Chain Amino Acid Catabolism and Mitigates Lung Metastasis of Adenoid Cystic Carcinoma. Adv Sci (Weinh). 2025;12(8):e2408751. https://doi.org/10.1002/advs.202408751

https://doi.org/10.1002/advs.202408751

Description of the errors:

In the original published paper, we found that the images of the “ITGA2^+^PDGFRα^+^” and “ITGA2^−^PDGFRα^+^” groups in Figure 4J were incorrectly inserted during the figure layout process. We tracked down the original data and have replaced Figure 4J with the correct image as follows:


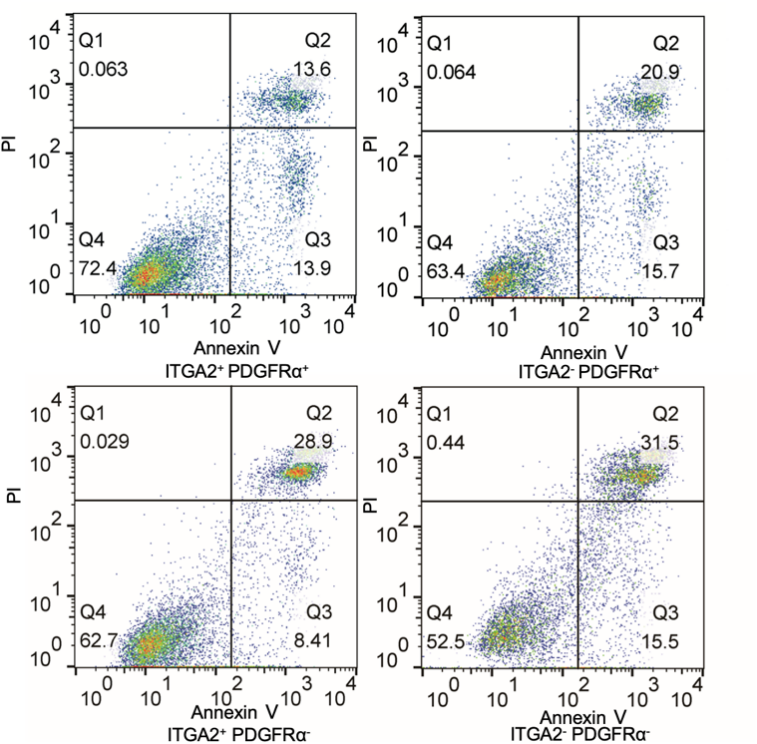


The analysis of Figure 4J was completed before the figure layout, and we have provided the analysis data to the editors. This correction will not affect other results or the conclusion of the study.

We apologize for this error.

