# Supplementary material for: Inhibition of CDH11 Activates cGAS‐STING by Stimulating Branched Chain Amino Acid Catabolism and Mitigates Lung Metastasis of Adenoid Cystic Carcinoma
Source: Adv Sci (Weinh). 2024 Dec 31;12(8):2408751. doi: 10.1002/advs.202408751 (PMC11848559; doi:10.1002/advs.202408751)
Supplement: Supplementary file 1 — Supporting Information [file ADVS-12-2408751-s001.docx]

Supporting Information

**Inhibition of CDH11 activates cGAS-STING by stimulating branched chain amino acids catabolism and mitigates lung metastasis of adenoid cystic carcinoma**

*Rui-Feng Li, Shuo Liu, Qian Gao, Min Fu, Xin-Yi Sun, Mian Xiao, Xi-Yuan Ge*****,*** *Xin Peng*********.*

*Corresponding author. Email: pxpengxin@263.net, gexiyuan@bjmu.edu.cn

**This file includes:**

Figure S1 to Figure S6
Supplementary Table 1 (separate file). Genes with CNV of SACC

Supplementary Table 2. Primers are used in this study

Figure S1.

Kyoto Encyclopedia of Genes and Genomes (KEGG) enriched signal pathways of cluster 0 to 7.


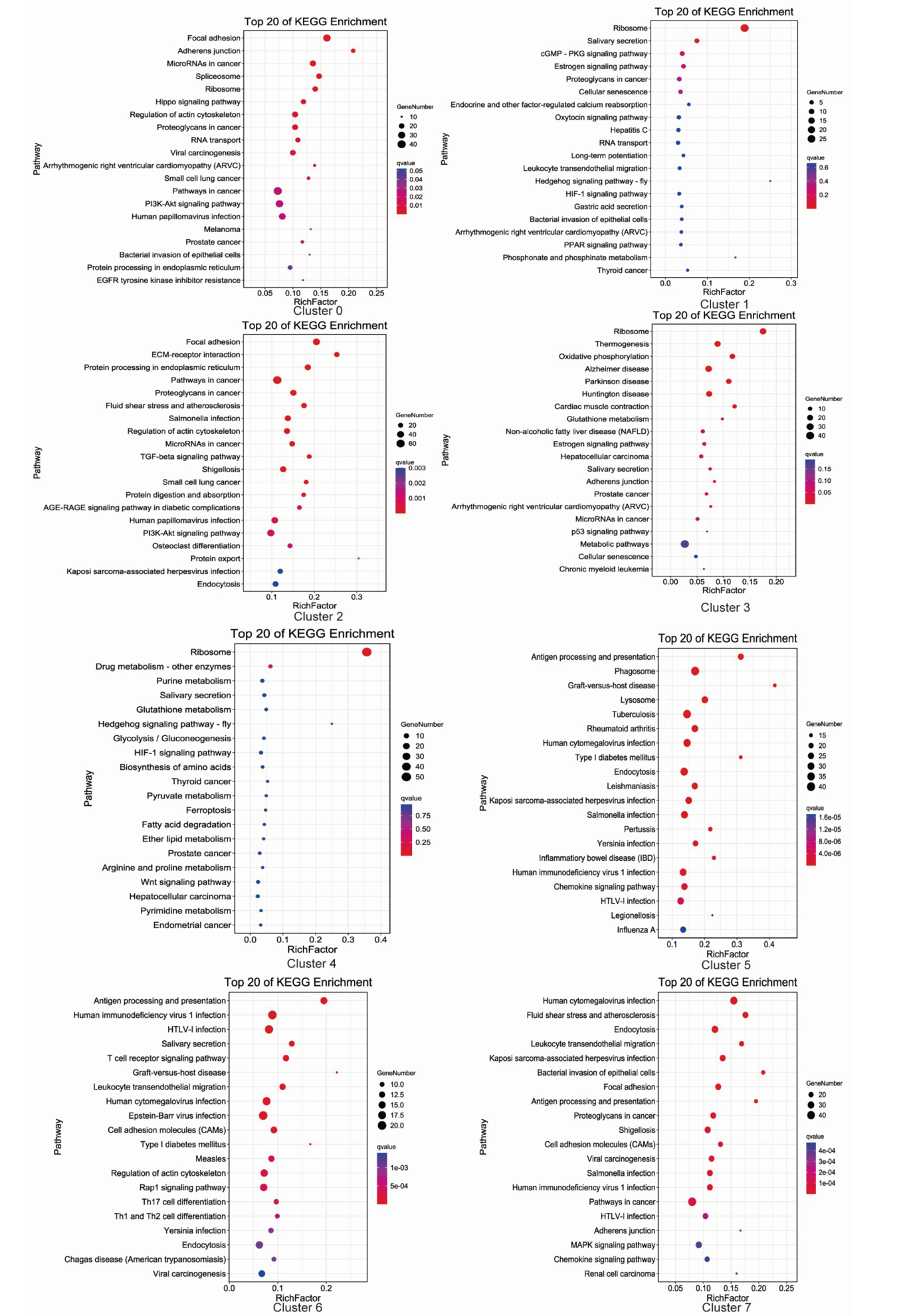


Fig. S2.

Kyoto Encyclopedia of Genes and Genomes (KEGG) enriched signal pathways of cluster 8 to 13.


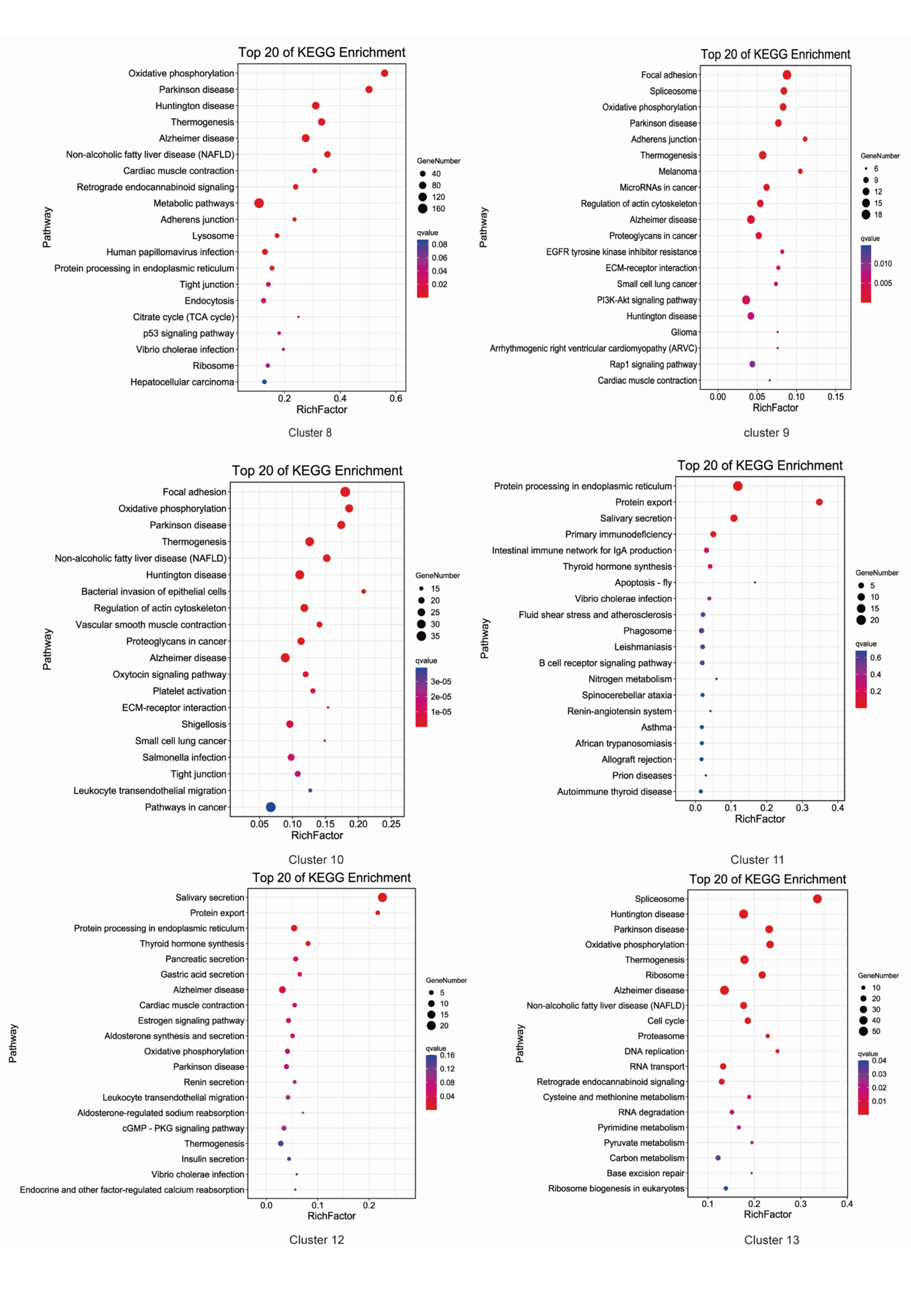


Fig. S3.

Kyoto Encyclopedia of Genes and Genomes (KEGG) enriched signal pathways of cluster 14 to 19 .


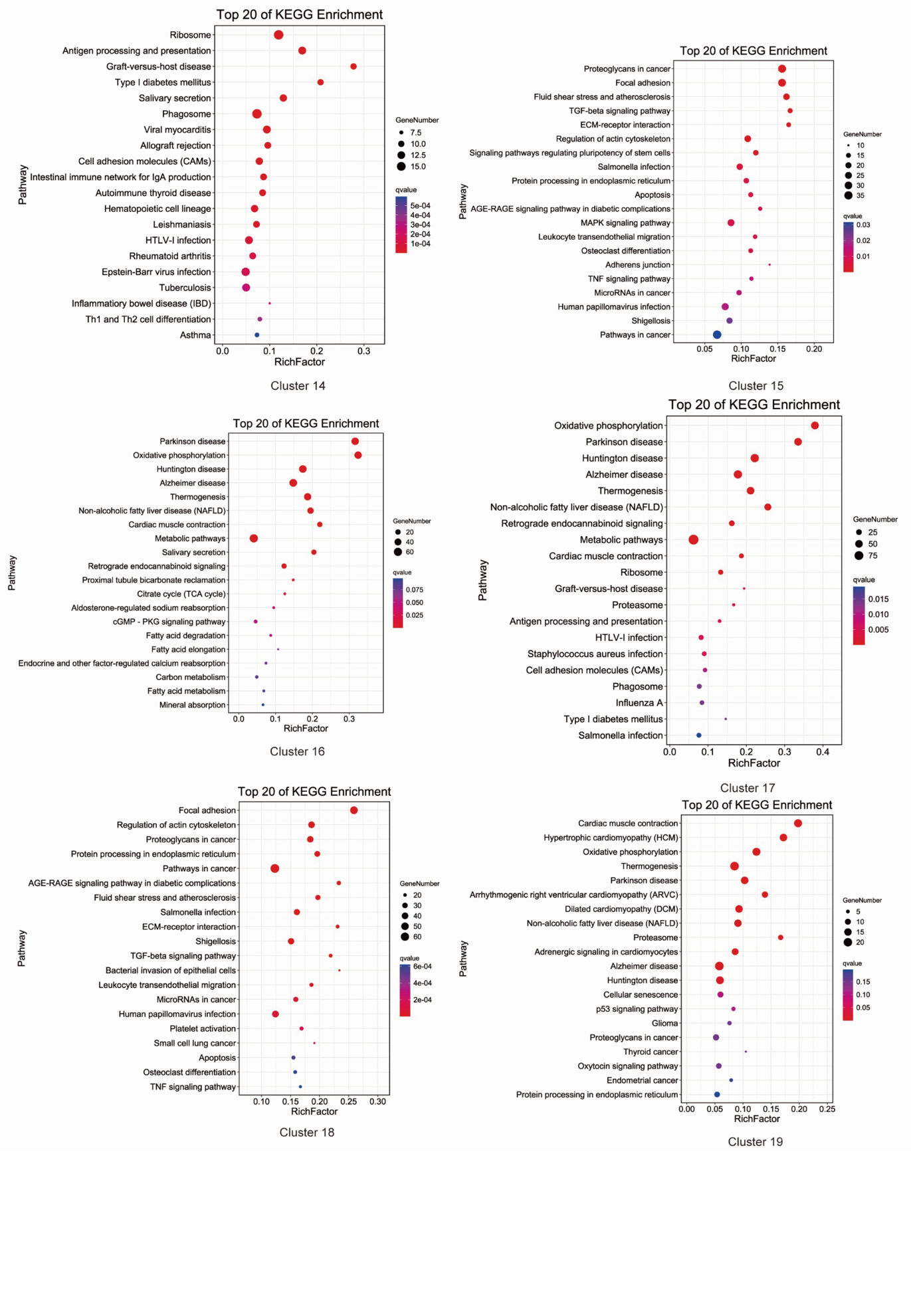


Fig. S4.

The hybrid EMT state cells reside in the vascular-fibrous microenvironment and exhibit a pro-tumorigenic impact.

(A) The UMAP of the NK and T cells was identified after reclustering. (B) The cell percentage frequency of NK and T cell subpopulations among different groups. (C) The UMAP of scRNA-seq cells recovered from both the SMG and SACC labeled by cluster. 18 clusters were identified after data combination and batch-effect correction. (D) The UMAP of scRNA-seq cells recovered from both SMG and SACC cells labeled by by patient sample. (E) The CNA scores of various subsets of epithelial cells. (F) Regulon activity distribution of different epithelial cell subsets. (G) Cell cycle analyses shows the percentage of cells in different cell cycle state of epithelial subpopulations. (H) qRT-PCR showed that VIM mRNA expressed level of flow cytometry sorting cells. *n* = 3, Means ± SEM are shown, * *P* < 0.05 using paired test. (I) The cellular morphology and quantity of clone formation assay. (J) Upper, Transwell assays were performed to assess cell migration, using crystal violet staining for both morphological changes and quantification purposes. Under, Cells were tested for their invasion ability using matrigel-coated Transwell assays.


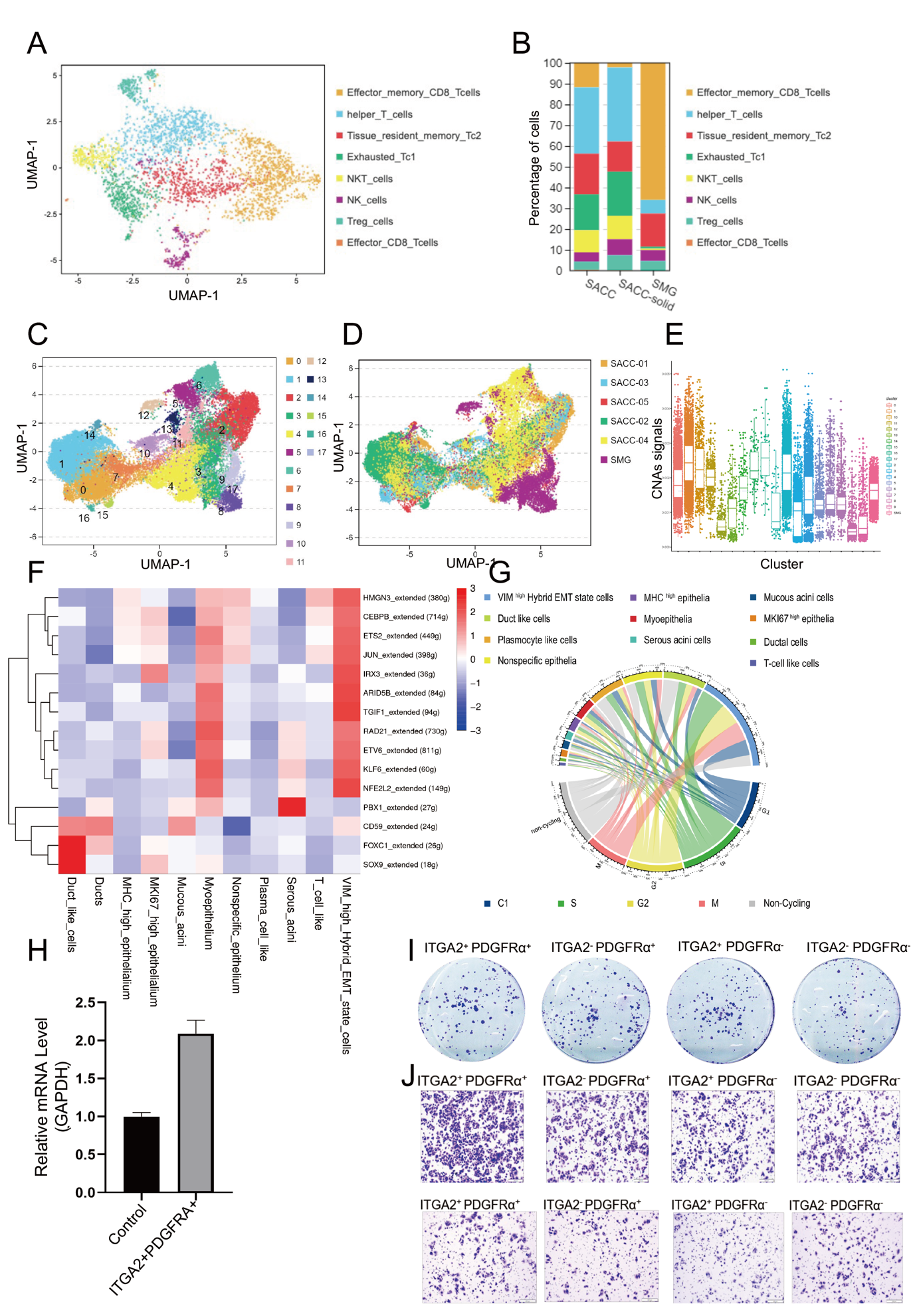


Fig. S5.

The CDH11 acts as a biomarker for hybrid EMT state cells and CAFs

**(A)** The mRNA relative expression of CDH11 was assessed in 10 pairs of SMG and SACC tissues. *n* = 3, Means ± SD are shown, * *P* < 0.05 using t test. (B) Kaplan-Meier curves of cumulative survival (*n* = 120) based on CDH11 expression. Statistical significance was determined by two-tailed unpaired t-test, * *P* < 0.05. **(C)** Left, the UMAP shows the expression distribution of CDH11 in all cells, and CDH11 was highly expressed in some epithelial cells and some fibroblasts. Middle, the UMAP shows the expression distribution of CDH11 in fibroblasts, and CDH11 was highly expressed in some fibroblasts. Right, the UMAP shows the expression distribution of CDH11 in fibroblasts of SMG, and CDH11 is not expressed in fibroblasts of SMG. **(F)** Venn diagrams showing the overlap of the identified proteins in two co-IP-MS experiments using anti-CDH11 antibody. **(G)** The protein interaction with CDH11 detected by mass spectrometry. Plot with STRING (https://cn.string-db.org/) **(H)** Quantification of western blots from CETSA. **(I)** Left, the quantification of DNA Comet assays in SACC-83 cells treated with CXB, DMC or SD-133. Means ± SEM are shown. **P* <0 .05 by one-way analysis of variance (ANOVA). Middle, the quantification of DNA Comet assays in SACC-83 cells treated with CDH11 over-expression. Means ± SEM are shown. **P* <0 .05 by t test. Right, the quantification of DNA Comet assays in SACC-83 cells treated with CDH11 knockdown. Means ± SEM are shown. * *P* > 0.05 using t test. More than 100 cells were analyzed per group.


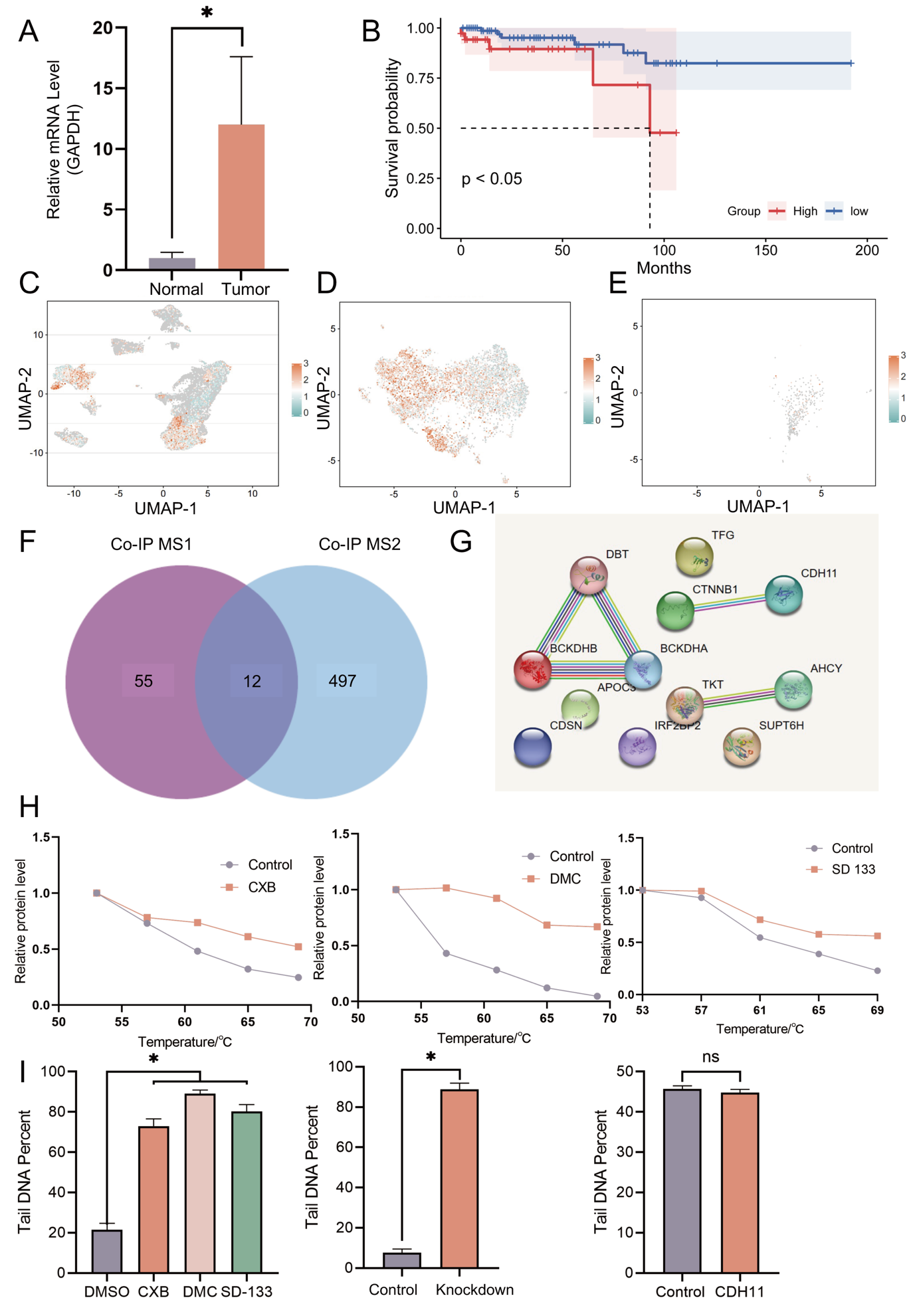


Fig. S6.

The binding of CXB/DMC/SD-133 to CDH11.

(A) The Recombinant Human His-Tagged Cadherin-11 Protein was immobilized on a CM7 Surface using the thiol coupling method. Wild type cadherin-11 was injected at various concentrations using the Biacore 1K Cytiva instrument. Each concentration underwent three injections, demonstrating excellent binding reproducibility. Colored lines represent experimental data-points, while black lines depict curve fits. The order is as follows: CXB, DMC and SD-133. (B) The prediction of the activity pocket for Recombinant Human His-Tagged Cadherin-11 Protein is depicted in red. (C) Upper, CXB with interactive residue side chains at the pocket are shown in stick rendering, with the inhibitors drawn in colorful. The polypeptide backbones are rendered as ribbons. Under, DMC with interactive residue side chains at the pocket are shown in stick rendering. The yellow broken lines indicate potential intermolecular hydrogen bonds, while the gray broken lines indicate pi-cation interactions. (D) Hematoxylin-eosin staining (H&E) staining of the heart, liver, spleen and kindey. (E) Flow analysis of CD8+ cells


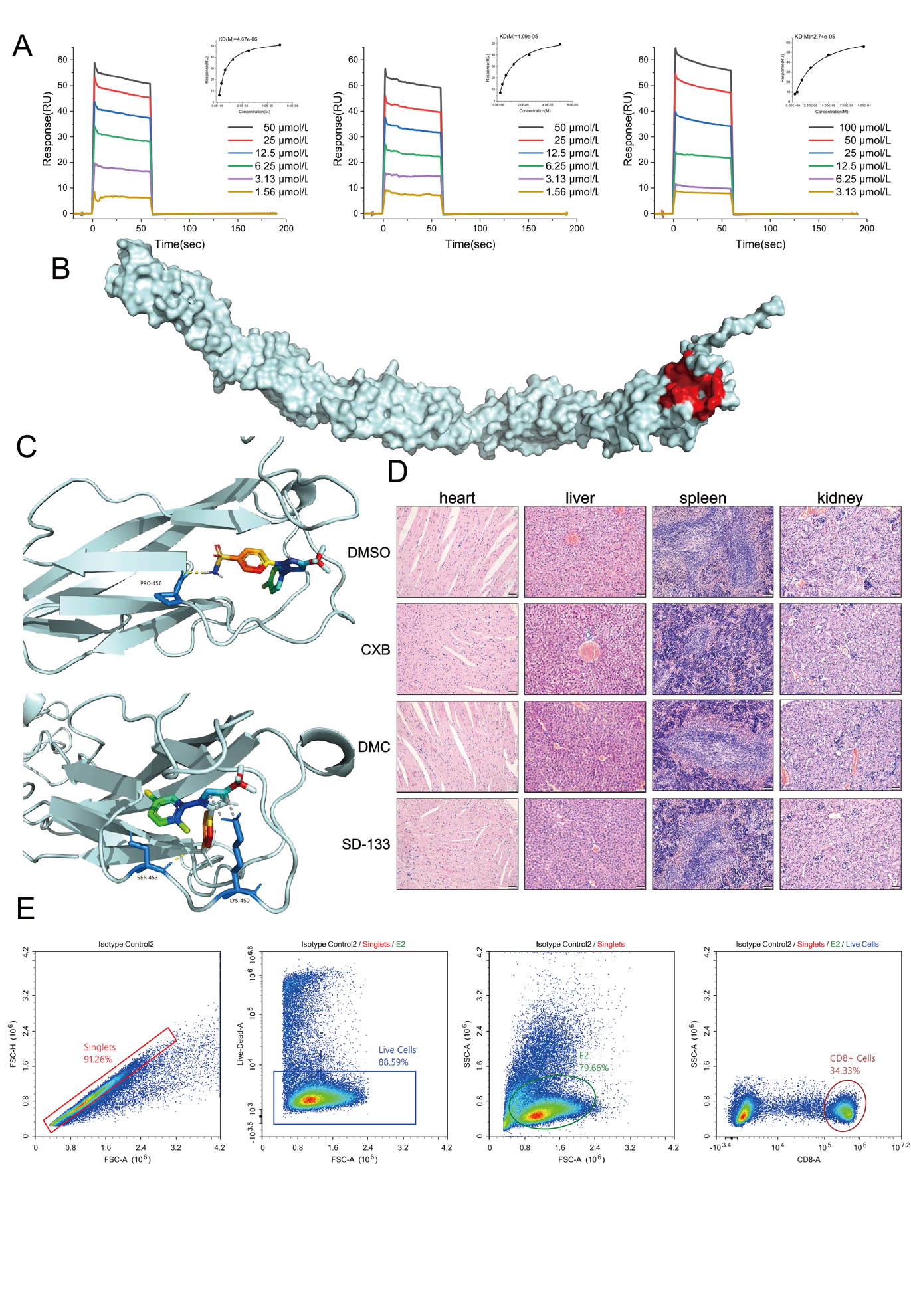


Supplementary Table 2. Primers are used in this study

| **Gene Name** | **Forward/Reverse** | **Sequence** |
| --- | --- | --- |
| CDH11 | Forward Sequence | GATCGTCACACTGACCTCGACA |
|  | Reverse Sequence | CTTTGGCTTCCTGATGCCGATTG |
| VM | Forward Sequence | AGGCAAAGCAGGAGTCCACTGA |
|  | Reverse Sequence | ATCTGGCGTTCCAGGGACTCAT |
| TWIST1 | Forward Sequence | GCCAGGTACATCGACTTCCTCT |
|  | Reverse Sequence | TCCATCCTCCAGACCGAGAAGG |
| SNAI1 | Forward Sequence | TGCCCTCAAGATGCACATCCGA |
|  | Reverse Sequence | GGGACAGGAGAAGGGCTTCTC |
| ZEB1 | Forward Sequence | GGCATACACCTACTCAACTACGG |
|  | Reverse Sequence | TGGGCGGTGTAGAATCAGAGTC |
| CDH1 | Forward Sequence | GCCTCCTGAAAAGAGAGTGGAAG |
|  | Reverse Sequence | TGGCAGTGTCTCTCCAAATCCG |
| CDH2 | Forward Sequence | CCTCCAGAGTTTACTGCCATGAC |
|  | Reverse Sequence | GTAGGATCTCCGCCACTGATTC |
| MMP2 | Forward Sequence | AGCGAGTGGATGCCGCCTTTAA |
|  | Reverse Sequence | CATTCCAGGCATCTGCGATGAG |
| IFNB1 | Forward Sequence | CTTGGATTCCTACAAAGAAGCAGC |
|  | Reverse Sequence | TCCTCCTTCTGGAACTGCTGCA |
| GAPDH | Forward Sequence | GTCTCCTCTGACTTCAACAGCG |
|  | Reverse Sequence | ACCACCCTGTTGCTGTAGCCAA |
